# Comprehensive metabolomics and transcriptomics analyses investigating the regulatory effects of different sources of dietary astaxanthin on the antioxidant and immune functions of commercial-sized rainbow trout

**DOI:** 10.3389/fimmu.2024.1408168

**Published:** 2024-09-26

**Authors:** Li Chen, Lei Wang, Yaopeng Li, Xugan Wu, Xiaowen Long

**Affiliations:** ^1^ College of Agriculture and Biological Science, Dali University, Dali, China; ^2^ Team for Aquatic Ecology in Erhai Lake Watershed, Dali University, Dali, China; ^3^ Co-Innovation Center for Cangshan Mountain and Erhai Lake Integrated Protection and Green Development of Yunnan Province, Dali University, Dali, China; ^4^ Key Laboratory of Freshwater Aquatic Genetic Resources, Ministry of Agriculture, Shanghai Ocean University, Shanghai, China; ^5^ National Experimental Teaching Demonstration Center of Aquatic Science, Shanghai Ocean University, Shanghai, China; ^6^ Research and Development (R & D) Center, Qinghai Minze Longyangxia Ecological Hydroponics Co., Ltd, Hainan, China

**Keywords:** astaxanthin, *Oncorhynchus mykiss*, metabolomics, transcriptomics, antioxidant capacity, immunity

## Abstract

Astaxanthin is an important aquatic feed additive that enhances the antioxidant capacity, and immune function of rainbow trout (*Oncorhynchus mykiss*); however, very limited information is available on its underlying molecular mechanisms. *Haematococcus pluvialis* powder, *Phaffia rhodozyma* powder, and synthetic astaxanthin were added to the commercial feed (no astaxanthin, NA) to prepare three experimental feeds, referred to as the HPA, PRA, and SA groups, respectively, and their actual astaxanthin contents were 31.25, 32.96, and 31.50 mg.kg^-1^, respectively. A 16-week feeding trial was conducted on the *O. mykiss* with an initial body weight of 669.88 ± 36.22 g. Serum and head kidney samples from commercial-sized *O. mykiss* were collected for metabolomics and transcriptomics analysis, respectively. Metabolomics analysis of the serum revealed a total of 85 differential metabolites between the astaxanthin-supplemented group and the control group. These metabolites were involved in more than 30 metabolic pathways, such as glycerophospholipid metabolism, fatty acid biosynthesis, linoleic acid metabolism, and arginine and proline metabolism. It is speculated that different sources of dietary astaxanthin may regulate antioxidant capacity and immunity mainly by affecting lipid metabolism and amino acid metabolism. Transcriptomic analysis of the head kidney revealed that the differentially expressed genes between the astaxanthin-supplemented group and the control group, such as integrin beta-1 (*ITGB1*), alpha-2-macroglobulin (*A2M*), diamine acetyltransferase 1 (*SAT1*), CCAAT/enhancer-binding protein beta (*CEBPB*) and DNA damage-inducible protein 45 alpha (*GADD45A*), which are involved in cell adhesion molecules, the *FoxO* signaling pathway, phagosomes, and arginine and proline metabolism and play regulatory roles in different stages of the antioxidant and immune response of *O. mykiss*.

## 1 Introduction

Rainbow trout (*Oncorhynchus mykiss*) is an important commercial aquaculture species that has been farmed extensively worldwide. It is popular among consumers because of its abundance of amino acids, minerals, vitamins, highly unsaturated fatty acids [such as eicosapentaenoic acid (EPA) and docosahexaenoic acid (DHA)], and other beneficial properties (1). Currently, rainbow trout are generally farmed in high-density or intensive ways, such as through cage culture and running water culture. However, these farming methods can increase fishery production but inevitably cause a series of problems. Previous studies have shown that infectious diseases caused by various bacterial and viral pathogens remain a major obstacle to rainbow trout farming and are often the leading cause of economic losses and restricting the development of aquaculture (2–4). Therefore, enhancing the immunity and disease resistance of rainbow trout is currently a significant concern for aquatic animal nutritionists.

Astaxanthin (3, 3’ - dihydroxy - β, β-carotene-4, 4’ - dione) is a keto-carotenoid that is widely found in microorganisms and marine organisms, such as shrimp, crab, salmon, trout, krill, microalgae, and yeast. It has strong antioxidant properties, as well as anti-inflammatory, anti-apoptotic, and immune-modulatory functions (5, 6). Therefore, it is generally used in food (7), biomedical science (8), aquatic feed (9), and other fields. At present, natural astaxanthin and synthetic astaxanthin are the two main sources of astaxanthin, of which natural astaxanthin sources mainly include green algae (*Haematococcus pluvialis*) (10), red yeast (*Phaffia rhodozyma*) (11), bacteria (*Paracoccus carotinifaciens*) (12) and Antarctic krill (*Euphausia superba*) (13). The price of astaxanthin from *H. pluvialis* is relatively high, while the price of astaxanthin from *P. rhodozyma* and *P. carotinifaciens* is moderate, and both have been used in the salmon aquaculture industry (12, 14).

Astaxanthin not only plays a role in regulating the color of salmon and trout, but also has important biological functions, such as antioxidant activity, immune response regulation and disease resistance in fish (6, 15, 16), and different physiological functions have been found among different sources of astaxanthin (11, 17–20). Previous studies on the effects of dietary supplementation with astaxanthin on the antioxidant and immune functions of salmon and trout have focused mainly on the activities of antioxidant and immune-related enzymes and their gene expression levels. These studies have shown that dietary *P. rhodozyma* astaxanthin could significantly reduce the activities of catalase (CAT), glutamic oxaloacetic transaminase (GOT), glutamic pyruvic transaminase (GPT) and lipid peroxide (LPO) in the plasma or serum of *O. mykiss* (21, 22), but increase the total carotenoid content, total antioxidant capacity (T-AOC) and superoxide dismutase (SOD) activity in plasma or serum (21, 23). Dietary supplementation with synthetic astaxanthin significantly increased the levels of antioxidants such as retinol, alpha-tocopherol, astaxanthin, and ascorbic acid in the muscle and liver of Atlantic salmon (*Salmo salar*) (24). In addition, supplementation with 100 mg.kg^-1^ synthetic astaxanthin in feed significantly enhanced the liver T-AOC of *O. mykiss* (25). However, relatively few studies have investigated the molecular regulatory effects of different sources (or different configurations) of astaxanthin on the antioxidant and immune functions of *O. mykiss*.

With the rapid development of next-generation sequencing (NGS) technology, it is possible to understand and characterize the molecular reactions of salmon and trout species in depth (26). Metabolomics is a new technology and discipline revealing the essence of metabolism; nuclear magnetic resonance (NMR), liquid chromatography-mass spectrometry (LC-MS), and gas chromatography-mass spectrometry (GC-MS) are generally used to qualitatively and quantitatively analyze small molecule metabolites in living organisms, and investigate the relationships between endogenous metabolites in response to internal or external factors (27, 28). Transcriptomics is a discipline that studies the transcription and regulation of genes in cells and is used to analyze the differences in the expression levels of certain genes in different tissues or different physiological states and to identify important functional genes or unknown genes with specific physiological functions (29, 30). At present, metabolomics and transcriptomics have been widely applied in studies of growth and development, disease resistance and immunity, phylogeny, and biotoxicological processes and related mechanisms in aquatic animals (31–34). For example, high-throughput sequencing technology has been used to systematically and comprehensively analyze fish immune response mechanisms (35), mine core immune-related gene clusters (36), and research immune response pathways (37).

This study was conducted to investigate the molecular regulatory effect of different sources of dietary astaxanthin on the antioxidant and immune functions of commercial-sized *O. mykiss* based on comprehensive metabolomics and transcriptomics analyses. These results could not only deepen our understanding of the molecular regulatory effects of different sources of astaxanthin on the antioxidant and immune functions of *O. mykiss* but also provide a reference for studies of antioxidant and immune functions in fish.

## 2 Materials and methods

### 2.1 Experimental diets

The commercial feed of rainbow trout was used as the basic diet (no astaxanthin, NA, crude protein: 42% dry diet; total lipids: 20% dry diet). Three different sources of astaxanthin (*H. pluvialis* powder, *P. rhodozyma* powder, and synthetic astaxanthin) were added to the basic diet to prepare three experimental diets; the theoretical content of dietary astaxanthin was 30 mg.kg^-1^, and the actual astaxanthin content was 31.25, 32.96, and 31.50 mg.kg^-1^, respectively (defined as the HPA, PRA, and SA groups, respectively). All experimental diets were processed by the Officially Aller Aqua (Qingdao) Co., Ltd., and sealed in a black bag, transported to the Qinghai Minze Longyangxia Ecological Aquatic Co., Ltd, Qinghai Province, China, and stored at -20°C for later use. The analysis of moisture, crude protein, and ash in experimental diets was conducted according to AOAC procedures (38). Total lipids were extracted with chloroform-methanol (2:1, *v/v*) according to the method described by Folch et al. (39). The nutrient compositions of the four experimental diets (NA, HPA, PRA, and SA) are shown in **Table 1**. The ingredients of experimental feeds mainly include fish meal, soybean meal, rapeseed meal, fish oil, soybean oil, soy lecithin, calcium dihydrogen phosphate, vitamin premix, and mineral premix. Since the feed formulations (the proportions of the ingredients) involved commercial confidentiality from the Officially Aller Aqua (Qingdao) Co., Ltd., this information could not be provided in this paper.

**Table 1:** Proximate composition and actual astaxanthin content of the four experimental diets.

Items	NA	HPA	PRA	SA
Moisture (% dry diet)	9.37	10.13	9.35	9.58
Crude protein (% dry diet)	42.37	42.02	42.85	42.81
Total lipids (% dry diet)	24.04	24.71	24.53	24.54
Ash (% dry diet)	6.91	6.88	7.03	6.80
Astaxanthin (mg.kg^-1^ dry diet)	0	31.25	32.96	31.50

NA, no astaxanthin; HPA, Haematococcus pluvialis astaxanthin; PRA, Phaffia rhodozyma astaxanthin; SA, synthetic astaxanthin.

### 2.2 Experimental design and culture management

This experiment was conducted at Qinghai Minze Longyangxia Ecological Aquatic Co., Ltd, Qinghai Province, China. A total of 1800 healthy and active *O. mykiss* juveniles with a body weight of 669.88 ± 36.22 g were selected and randomly stocked into 12 net cages (length × width × height = 4 m × 4 m × 5 m). There were four diet groups (defined as NA, HPA, PRA, and SA), with each group having three replicate net cages (150 fish per net cage). Before the feeding trial, all the *O. mykiss* were acclimated to the culture conditions for 1 week and fed the NA diet (without astaxanthin). During the feeding trial, the water temperature ranged from 12-19°C, and the dissolved oxygen content ranged from 6-9 mg. L^-1^, and the water transparency was approximately 2.50 m; all the fish were fed to apparent satiation twice (at 8:00 and 16:00) daily for 4 months.

### 2.3 Sample collection

At the end of the feeding trial, the fish were fasted for 24 hours before sampling. Three fish were selected randomly and anesthetized with 30 mg. L^-1^ eugenol from each net cage; therefore, six fish were selected for each diet group. Approximately 10 mL of blood was drawn from the caudal vein with a syringe. The blood samples were centrifuged at 5, 000 × *g* for 10 min at 4°C, after which the supernatant (serum) was collected, divided into several aliquots, snap-frozen in liquid nitrogen, and stored at -80°C for subsequent metabolomics analysis. When metabolomics analysis was performed, a total of 6 fish serum samples were taken from each diet group. The rainbow trout were dissected, and the head kidney tissues were sampled and put into a 2 mL cryotube. Then 1.5 mL of RNAlater (Cat.R0901, Merck Company, Germany) was added, and the samples were stored at -80°C for subsequent transcriptomic analysis.

### 2.4 Serum metabolomics analysis

One hundred microliters of serum in a 1.5 mL Eppendorf tube was mixed with 300 μL of methanol-acetonitrile (2:1, *v/v*) and 10 μL of L-2-chlorophenylalanine (0.3 mg.mL^-1^ stock in dH_2_O, as an internal quantitative standard) and vortexed for 1 min by a vortex mixer (QL-901, Jiangsu Haimen Qilin Beier Instrument Manufacturing Co., Ltd., Haimen, China) before being placed in -20°C freezer for 30 min. The mixture was then centrifuged at 13000 r·min^-1^ and 4°C for 10 min (Eppendorf 5417R, Eppendorf Co., Hamburg, Germany); subsequently, 200 μL of the supernatant was collected into a 2 mL HPLC glass vial and blown dry with nitrogen, and 300 μL of methanol-water (1:4, *v/v*) was added to each sample and vortexed for 30 s and placed at 20°C for 2 h. The mixture was then centrifuged at 13000 r·min^-1^ at 4°C for 10 min, after which 150 μL of the supernatant was filtered with a syringe through a 0.22 μm organic phase pinhole filter membrane, and collected into a 2 mL HPLC glass vial for later analysis.

The metabolites in the serum were analyzed using a liquid-mass spectrometry system consisting of a Nexera UPLC ultra-high performance liquid chromatography-tandem QE high-resolution mass spectrometer. The chromatography was performed on an ACQUITY UPLC HSS T3 (100 mm × 2.1 mm, 1.8 μm). A stepwise elution program with two mobile phases, A-water (containing 0.1% formic acid), and B-acetonitrile (containing 0.1% formic acid), was used (**Supplementary Table 1**). The flow rate was 0.35 mL·min^-1^ and the column temperature was 45°C. The injection volume was 2 μL. An electrospray ion source (ESI) was used as the ion source. The positive and negative ion scanning modes were used to collect the sample quality spectrum signals. The mass spectrum parameters are shown in **Supplementary Table 2**.

### 2.5 Head kidney transcriptomics analysis

#### 2.5.1 Total RNA extraction

Total RNA in the head kidney was extracted using TRIzol reagent (TaKaRa, Dalian, China), and the concentration and purity of total RNA were detected and assessed by Nanodrop 2000 (Thermo Scientific, USA) and agarose gel electrophoresis, respectively. RNA integrity was assessed using an Agilent 2100 Bioanalyzer (Agilent Technologies, Santa Clara, CA, USA). Subsequently, cDNA libraries were constructed using TruSeq Stranded mRNA LT Sample Prep Kit (Illumina, San Diego, CA, USA) according to the manufacturer’s instructions. Then, these libraries were sequenced on the Illumina sequencing platform (HiSeqTM 2500 or Illumina HiSeq X Ten), and 125 bp/150 bp paired-end reads were generated. The transcriptome sequencing and analysis were conducted by OE Biotech Co., Ltd. (Shanghai, China).

#### 2.5.2 *De novo* assembly and analysis of the transcriptome

The raw data (raw reads) in fastq format were first processed using Trimmomatic and the low-quality reads were removed to obtain the clean reads. The clean reads were mapped to the reference genome using HISAT2. Position information on the reference genome or gene, as well as sequence feature information unique to the sequenced sample, was obtained. The FPKM of each gene was calculated using Cufflinks, and the read counts of each gene were obtained by HTSeqcount. Differential expression analysis was performed using the DESeq (2012) R package. A P value < 0.05 and fold change > 2 or < 0.5 were set as the thresholds for significantly differential expression. Enrichment analysis was performed on differentially expressed genes (DEGs) to determine the biological functions or pathways affected by the DEGs.

#### 2.5.3 Validation of the RNA-seq profiles by quantitative real-time PCR

Four randomly selected DEGs identified by transcriptome sequencing analysis were used for RNA-seq validation via qRT-PCR. First-strand cDNA synthesis was performed with a Prime-Script™ 1st Strand cDNA Synthesis Kit (RR036A, Takara Bio, Japan) following the manufacturer’s instructions. The qRT-PCR assays were performed with three replicates, and the β-actin gene was used as an internal control to normalize the expression level of the target genes. Specific primers were designed according to the unigene sequences using Primer 5.0 software (**Supplementary Table 3**). Gene expression levels were assessed using SYBR® Premix Ex Taq (TaKaRa) in a 20 μL reaction with a QuantStudio 3 real-time PCR instrument. The relative gene expression was calculated by 2^−△△Ct^ method. The results were analyzed by Student’s t-test using SPSS 16.0 software, and the significance level was set at *P* < 0.05.

## 3 Results

### 3.1 Serum metabolomics analysis

#### 3.1.1 Serum metabolic profile and comparison

**Figure 1:**
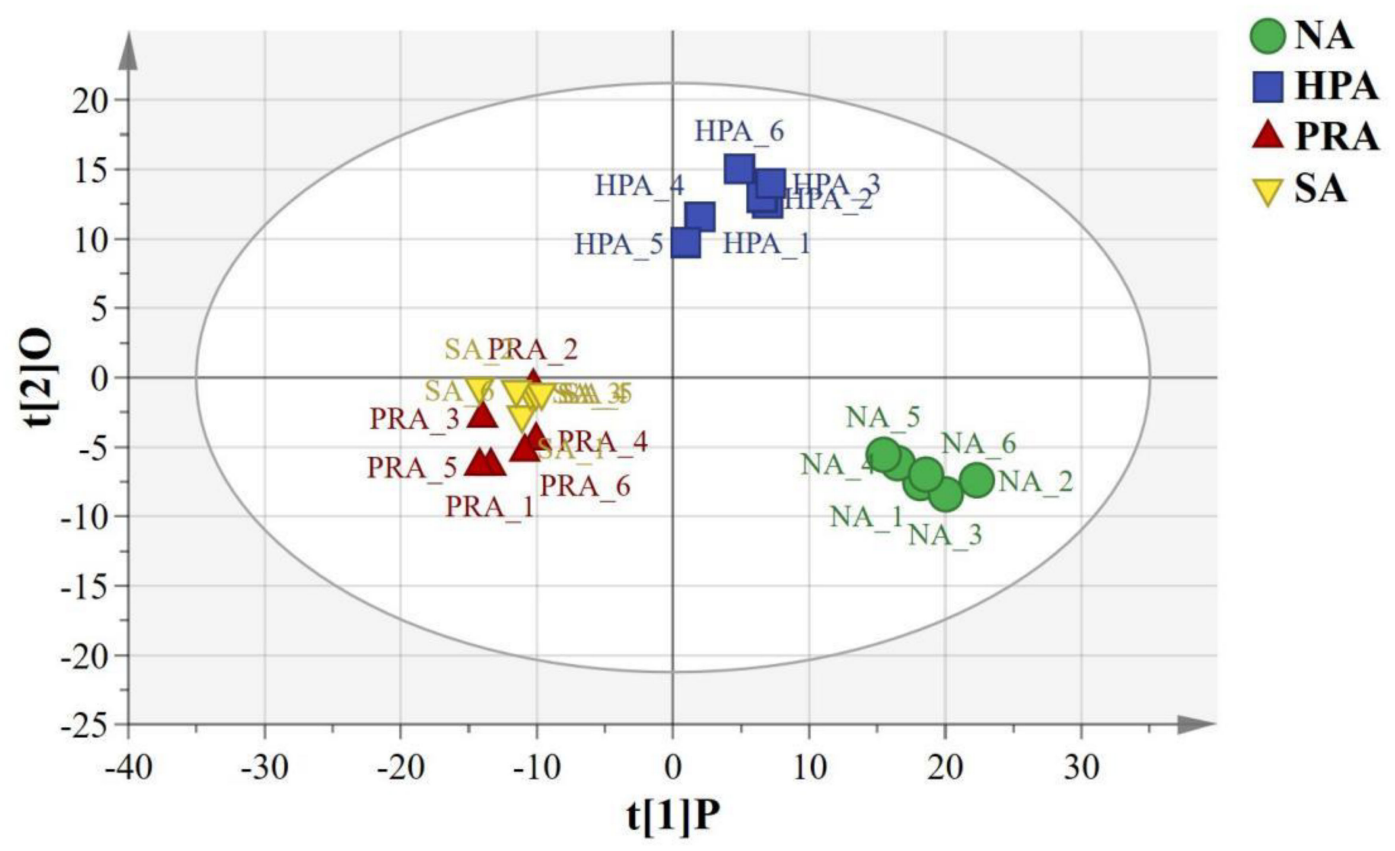
OPLS-DA score plots based on the LC-MS spectra of serum samples from *O. mykiss* among the different sources of dietary astaxanthin groups. “O” means “Orthogonal” and “P” means “Predictive” in OPLS-DA. R^2^X [1] =0.174, R^2^Y [2]= 0.0637, Ellipse: Hotelling’s T^2^ (95%). NA, no astaxanthin; HPA, *Haematococcus pluvialis* astaxanthin; PRA, *Phaffia rhodozyma* astaxanthin; SA, synthetic astaxanthin.

The orthogonal partial least squares discriminant analysis (OPLS-DA) of the LC-MS metabolic profiles of the serum showed significantly separated clusters between the NA and the HPA groups, between the NA and the PRA groups, between the NA and SA groups, and between the HPA and the PRA and the SA groups, respectively, and all the samples from the four diet groups were within the 95% Hotelling’s T^2^ ellipse (**Figure 1**).

The total number of metabolites detected within the serum was 983. There were 11 significantly differential metabolites in the serum between the NA and HPA groups, among which 8 metabolites were up-regulated and 3 metabolites were down-regulated in the HPA group compared with the NA group (**Supplementary Table 4**). There were 15 differential metabolites in the serum of *O. mykiss* between the NA and PRA groups, among which 11 metabolites were up-regulated and 5 metabolites were down-regulated in the PRA group (**Supplementary Table 5**). There were 18 differential metabolites in the serum of *O. mykiss* between the NA and SA groups, among which 12 metabolites were up-regulated and 5 metabolites were down-regulated in the SA group (**Supplementary Table 6**). There were 21 differential metabolites in the serum of *O. mykiss* between the HPA and PRA groups, among which 11 metabolites were up-regulated and 10 metabolites were down-regulated in the PRA group (**Supplementary Table 7**). There were 18 differential metabolites in the serum of *O. mykiss* between the HPA and SA groups, among which 10 metabolites were up-regulated and 8 metabolites were down-regulated in the SA group (**Supplementary Table 8**). These differential metabolites among the four diet groups mainly included phosphatidylcholine (PC), phosphatidylethanolamine (PE), phosphatidylinositol (PI), LysoPC, palmitic acid and palmitoleic acid, aniline, glycyl-glycine, L-histidine, sphingosine, etc.

#### 3.1.2 Serum metabolic pathway analysis

The differential metabolites identified in the serum between the NA and HPA groups were mainly involved in glycerophospholipid metabolism, fatty acid biosynthesis, autophagy-other, autophagy-animal, glycosylphosphatidylinositol (GPI)-anchor biosynthesis, linoleic acid metabolism, fatty acid elongation, alpha-linolenic acid metabolism, galactose metabolism, histidine metabolism, and fatty acid degradation (**Table 2**). The differential metabolites identified in the serum between the NA and PRA groups were mainly involved in glycerophospholipid metabolism, alpha-linolenic acid metabolism, autophagy-other, autophagy-animal, glycosylphosphatidylinositol (GPI)-anchor biosynthesis, linoleic acid metabolism, neuroactive ligand-receptor interaction, fatty acid biosynthesis, and arachidonic acid metabolism (**Table 3**). The differential metabolites identified in the serum between the NA and SA groups were mainly involved in histidine metabolism, glycerophospholipid metabolism, D-arginine and D-ornithine metabolism, linoleic acid metabolism, neuroactive ligand-receptor interaction, alpha-linolenic acid metabolism, fatty acid biosynthesis, phenylalanine metabolism, arachidonic acid metabolism, arginine and proline metabolism, and drug metabolism - cytochrome P450 (**Table 4**). The differential metabolites identified in the serum between the HPA and PRA groups were mainly involved in histidine metabolism, glycerophospholipid metabolism, apoptosis, necroptosis, D-arginine and D-ornithine metabolism, sphingolipid metabolism, linoleic acid metabolism, alpha-linolenic acid metabolism, galactose metabolism, phenylalanine metabolism, biosynthesis of unsaturated fatty acids, arachidonic acid metabolism, and arginine and proline metabolism (**Table 5**). The differential metabolites identified in the serum between the HPA and SA groups were mainly involved in the biosynthesis of unsaturated fatty acids, histidine metabolism, D-arginine and D-ornithine metabolism, D-glutamine and D-glutamate metabolism, nitrogen metabolism, arginine biosynthesis, alanine, aspartate and glutamate metabolism, vitamin B6 metabolism, fatty acid elongation, galactose metabolism, fatty acid degradation, glycine, serine and threonine metabolism, aminoacyl-tRNA biosynthesis, glycerophospholipid metabolism, drug metabolism-other enzymes, fatty acid biosynthesis, phenylalanine metabolism, glyoxylate and dicarboxylate metabolism, pyrimidine metabolism, arginine and proline metabolism, ABC transporters, purine metabolism, and steroid hormone biosynthesis (**Table 6**). These differential metabolites between the different diet groups were mainly related to fatty acid metabolism and amino acid metabolism.

**Table 2:** Metabolic pathways identified from the significantly different metabolites in the serum of *O. mykiss* between the NA and HPA groups.

Metabolic pathway	Significantly different metabolites ^a^
Glycerophospholipid metabolism	(0.31) PE (20:3(8Z,11Z,14Z)/22:6(4Z,7Z,10Z,13Z,16Z,19Z)) (12.37) PC(18:1(11Z)/18:1(11Z))
Fatty acid biosynthesis	(3.58) Palmitoleic acid (2.56) Palmitic acid
Autophagy - other	(0.31) PE (20:3(8Z,11Z,14Z)/22:6(4Z,7Z,10Z,13Z,16Z,19Z))
Autophagy - animal	(0.31) PE (20:3(8Z,11Z,14Z)/22:6(4Z,7Z,10Z,13Z,16Z,19Z))
Glycosylphosphatidylinositol (GPI)-anchor biosynthesis	(0.31) PE (20:3(8Z,11Z,14Z)/22:6(4Z,7Z,10Z,13Z,16Z,19Z))
Linoleic acid metabolism	(12.37) PC(18:1(11Z)/18:1(11Z))
Fatty acid elongation	(2.56) Palmitic acid
alpha-Linolenic acid metabolism	(12.37) PC(18:1(11Z)/18:1(11Z))
Galactose metabolism	(1.24) gamma-Glutamylleucine
Histidine metabolism	(0.85) Hydantoin-5-propionic acid
Fatty acid degradation	(2.56) Palmitic acid

a The number in parentheses is the fold change, mean contents of metabolites obtained from the HPA group/mean contents of metabolites obtained from the NA group. If the FC value is > 1, it means that metabolites in the HPA are more than in the NA.

**Table 3:** Metabolic pathways identified from the significantly different metabolites in the serum of *O. mykiss* between the NA and PRA groups.

Metabolic pathway	Significantly different metabolites ^a^
Glycerophospholipid metabolism	(0.22) PE(20:3(8Z,11Z,14Z)/22:6(4Z,7Z,10Z,13Z,16Z,19Z)) (20.04) PC(18:1(11Z**)/**18:1(11Z)) (0.38) LysoPC(20:0/0:0)
alpha-Linolenic acid metabolism	(2.11) (3Z,6Z)-3,6-Nonadienal (20.04) PC(18:1(11Z**)/**18:1(11Z))
Histidine metabolism	(0.31) Histamine; (20.24) L-Histidinol
Autophagy - other	(0.22) PE(20:3(8Z,11Z,14Z)/22:6(4Z,7Z,10Z,13Z,16Z,19Z))
Autophagy - animal	(0.22) PE(20:3(8Z,11Z,14Z)/22:6(4Z,7Z,10Z,13Z,16Z,19Z))
Glycosylphosphatidylinositol (GPI)-anchor biosynthesis	(0.22) PE(20:3(8Z,11Z,14Z)/22:6(4Z,7Z,10Z,13Z,16Z,19Z))
Linoleic acid metabolism	(20.04) PC(18:1(11Z**)/**18:1(11Z))
Neuroactive ligand-receptor interaction	(0.31) Histamine
Fatty acid biosynthesis	(4.64) Palmitoleic acid
Arachidonic acid metabolism	(20.04) PC(18:1(11Z**)/**18:1(11Z))

a The number in parentheses is the fold change, mean contents of metabolites obtained from the PRA group/mean contents of metabolites obtained from the NA group. If the FC value is > 1, it means that metabolites in the PRA are more than in the NA.

**Table 4:** Metabolic pathways identified from the significantly different metabolites in the serum of *O. mykiss* between the NA and SA groups.

Metabolic pathway	Significantly different metabolites ^a^
Histidine metabolism	(0.32) Histamine (19.86) L-Histidinol
Glycerophospholipid metabolism	(0.52) LysoPC(16:1(9Z)/0:0) (14.83) PC(18:1(11Z)/18:1(11Z))
D-arginine and D-ornithine metabolism	(33.58) 2-Oxoarginine
Linoleic acid metabolism	(14.83) PC(18:1(11Z)/18:1(11Z))
Neuroactive ligand-receptor interaction	(0.32) Histamine
alpha-Linolenic acid metabolism	(14.83) PC(18:1(11Z)/18:1(11Z))
Fatty acid biosynthesis	(3.69) Palmitoleic acid
Phenylalanine metabolism	(1.46) m-Coumaric acid
Arachidonic acid metabolism	(14.83) PC(18:1(11Z)/18:1(11Z))
Arginine and proline metabolism	(33.58) 2-Oxoarginine
Drug metabolism - cytochrome P450	(2.88) 2-ene-Valproic acid

a The number in parentheses is the fold change, mean contents of metabolites obtained from the SA group/mean contents of metabolites obtained from the NA group. If the FC value is > 1, it means that metabolites in the SA are more than in the NA.

**Table 5:** Metabolic pathways identified from the significantly different metabolites in the serum of *O. mykiss* between the HPA and PRA groups.

Metabolic pathway	Significantly different metabolites ^a^
Histidine metabolism	(1.20) Hydantoin-5-propionic acid (3.91) L-Histidinol
Glycerophospholipid metabolism	(1.62) PC(18:1(11Z)/18:1(11Z)) (0.67) LysoPC(18:3(6Z,9Z,12Z)) (0.72) LysoPC(22:5(7Z,10Z,13Z,16Z,19Z))
Apoptosis	(0.62) Sphingosine
Necroptosis	(0.62) Sphingosine
D-arginine and D-ornithine metabolism	(5.17) 2-Oxoarginine
Sphingolipid metabolism	(0.62) Sphingosine
Linoleic acid metabolism	(1.62) PC(18:1(11Z)/18:1(11Z))
alpha-Linolenic acid metabolism	(1.62) PC(18:1(11Z)/18:1(11Z))
Galactose metabolism	(0.29) Stachyose
Phenylalanine metabolism	(1.18) 2-Phenylacetamide
Biosynthesis of unsaturated fatty acids	(0.49) Arachidic acid
Arachidonic acid metabolism	(1.62) PC(18:1(11Z)/18:1(11Z))
Arginine and proline metabolism	(5.17) 2-Oxoarginine

a The number in parentheses is the fold change, mean contents of metabolites obtained from the PRA group/mean contents of metabolites obtained from the HPA group. If the FC value is > 1, it means that metabolites in the PRA are more than in the HPA.

**Table 6:** Metabolic pathways identified from the significantly different metabolites in the serum of *O. mykiss* between the HPA and SA groups.

Metabolic pathway	Significantly different metabolites ^a^
Biosynthesis of unsaturated fatty acids	(0.40) Arachidic acid (0.41) Behenic acid (0.57) Palmitic acid
Histidine metabolism	(1.19) Hydantoin-5-propionic acid (3.83) L-Histidinol
D-arginine and D-ornithine metabolism	(5.52) 2-Oxoarginine
D-glutamine and D-glutamate metabolism	(7.56) L-Glutamine
Nitrogen metabolism	(7.56) L-Glutamine
Arginine biosynthesis	(7.56) L-Glutamine
Alanine, aspartate and glutamate metabolism	(7.56) L-Glutamine
Vitamin B6 metabolism	(7.56) L-Glutamine
Fatty acid elongation	(0.57) Palmitic acid
Galactose metabolism	(0.49) Stachyose
Fatty acid degradation	(0.57) Palmitic acid
Glycine, serine and threonine metabolism	(270.84) L-2,4-diaminobutyric acid
Aminoacyl-tRNA biosynthesis	(7.56) L-Glutamine
Glycerophospholipid metabolism	(0.63) LysoPC(16:1(9Z)/0:0)
Drug metabolism - other enzymes	(7.87) Acetylisoniazid
Fatty acid biosynthesis	(0.57) Palmitic acid
Phenylalanine metabolism	(1.17) 2-Phenylacetamide
Glyoxylate and dicarboxylate metabolism	(7.56) L-Glutamine
Pyrimidine metabolism	(7.56) L-Glutamine
Arginine and proline metabolism	(5.52) 2-Oxoarginine
ABC transporters	(7.56) L-Glutamine
Purine metabolism	(7.56) L-Glutamine
Steroid hormone biosynthesis	(0.45) Estrone glucuronide

a The number in parentheses is the fold change, mean contents of metabolites obtained from the SA group/mean contents of metabolites obtained from the HPA group. If the FC value is > 1, it means that metabolites in the SA are more than in the HPA.

### 3.2 Head kidney transcriptomics analysis

#### 3.2.1 Transcriptome sequence assessment and annotation

A total of 86.15 G of clean reads were obtained from the head kidney transcriptome sequencing of the four diet groups after removing adaptor sequences and low-quality reads (**Table 7**). The effective amount of each sample was 6.72-7.5 G, the Q30 base content was 90.77-94.21%, and the average GC content was 49.46%. Clean reads were compared to the reference genome of the species using HISAT2, and 88.61-90.14% of the genome was compared for each sample.

**Table 7:** Raw reads and quality control of the *O. mykiss* cDNA libraries.

Samples	Number of raw reads (million reads)	Number of clear reads (million reads)	GC (%)	Q30 (%)
NA_1	49.50	48.50	49.61	90.93
NA_2	50.59	49.62	49.47	93.20
NA_3	51.68	50.66	49.37	92.83
HPA_1	47.69	46.79	49.52	92.88
HPA_2	50.11	49.06	49.67	93.23
HPA_3	51.65	50.72	49.30	93.12
PRA_1	51.63	50.67	49.58	93.54
PRA_2	50.01	49.22	49.40	94.21
PRA_3	47.81	46.05	49.30	90.84
SA_1	48.51	46.71	49.30	90.77
SA_2	47.43	45.69	49.59	90.98
SA_3	51.64	49.74	49.40	90.87

NA, no astaxanthin; HPA, Haematococcus pluvialis astaxanthin; PRA, Phaffia rhodozyma astaxanthin; SA, synthetic astaxanthin.

#### 3.2.2 Analysis of differentially expressed genes

**Figure 2:**
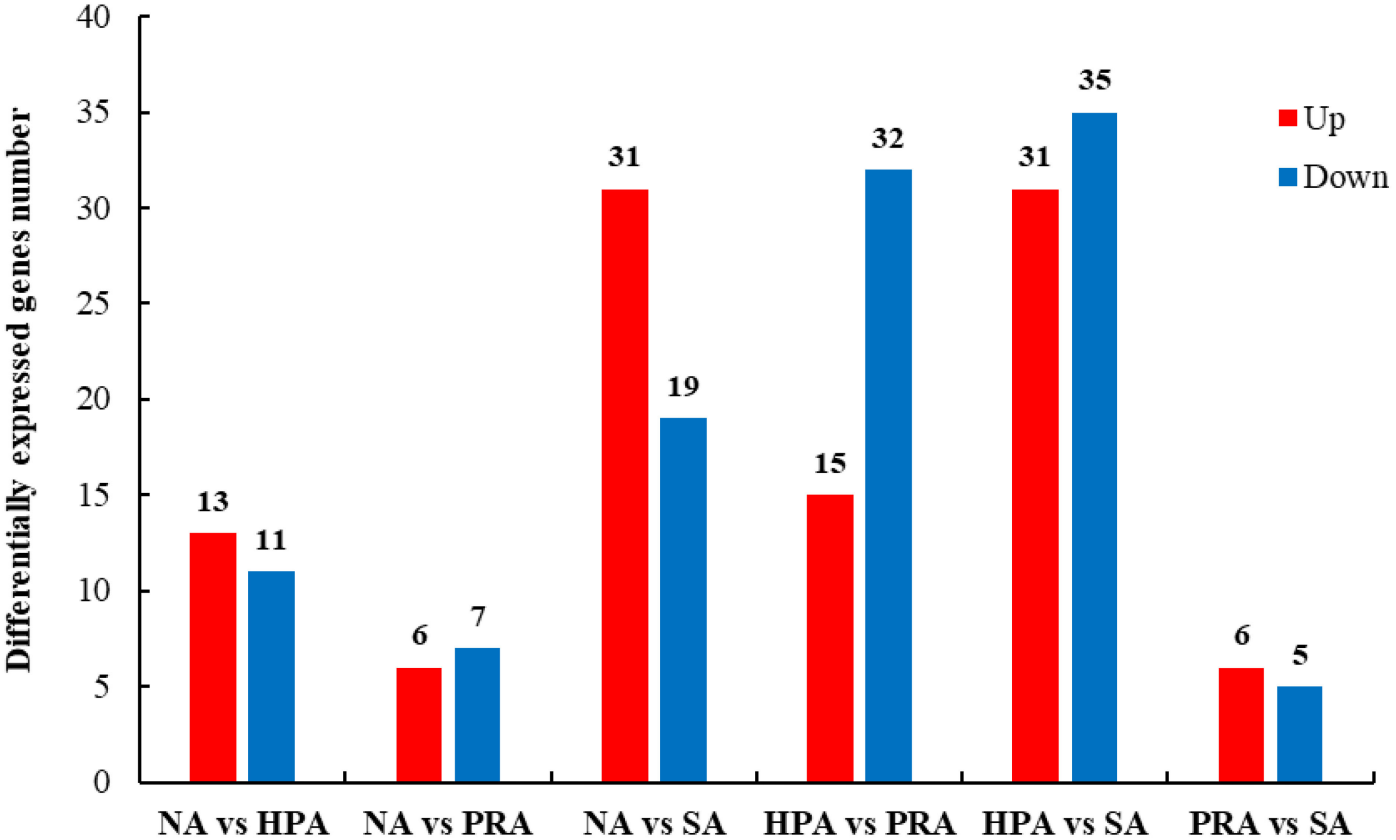
Summary of differentially expressed genes (DEGs) in the transcriptome of the head kidney of *O. mykiss* among different diet groups. NA, no astaxanthin; HPA, *Haematococcus pluvialis* astaxanthin; PRA, *Phaffia rhodozyma* astaxanthin; SA, synthetic astaxanthin.

A total of 211 differentially expressed genes (DEGs) in the head kidney were identified among the four diet groups (**Figure 2**). There were 24 DEGs in the head kidney of *O. mykiss* between the NA and HPA groups, among which 13 DEGs were up-regulated and 11 DEGs were down-regulated in the HPA group. These DEGs mainly included interleukin-2 receptor subunit beta, hemoglobin subunit beta-1, immunoglobulin kappa variable 1-33, and perforin-1. There were 13 DEGs in the head kidney of *O. mykiss* between the NA and PRA groups, among which 6 DEGs were up-regulated and 7 DEGs were down-regulated in the PRA group. These DEGs included mainly alpha-2-macroglobulin and integrin beta-1. There were 50 DEGs in the head kidney of *O. mykiss* between the NA and SA groups, among which 31 DEGs were up-regulated and 19 DEGs were down-regulated in the SA group. These DEGs mainly included interferon-induced protein 44-like, CXC chemokine receptor type 2, major histocompatibility complex class I-related gene protein, and tumor necrosis factor alpha-induced protein 2.

#### 3.2.3 GO functional annotation analysis of differentially expressed genes

**Figure 3:**
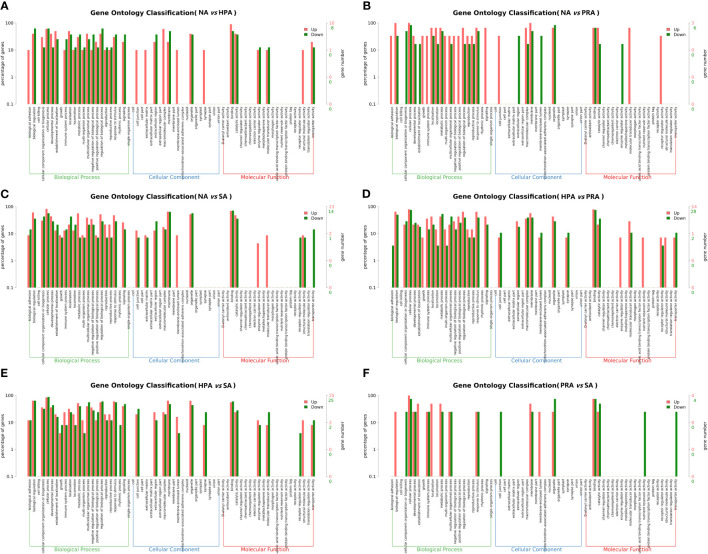
GO enrichment analyses of differentially expressed genes (DEGs) in the head kidney among different diet groups. **(A)** NA *vs* HPA; **(B)** NA *vs* PRA; **(C)** NA *vs* SA; **(D)** HPA *vs* PRA; **(E)** HPA *vs* SA; **(F)** PRA *vs* SA. NA, no astaxanthin; HPA, *Haematococcus pluvialis* astaxanthin; PRA, *Phaffia rhodozyma* astaxanthin; SA, synthetic astaxanthin.

To understand the functions of these DEGs, gene ontology (GO) assignments were conducted. A total of 211 DEGs were annotated to 1462 GO terms, among which 795 were annotated to biological processes, 369 were annotated to molecular functions, and 298 were annotated to cellular components. The DEGs between the NA and HPA groups (**Figure 3A**), the NA and PRA groups (**Figure 3B**), the NA and SA groups (**Figure 3C**), the HPA and PRA groups (**Figure 3D**), the HPA and SA groups (**Figure 3E**), the PRA and SA groups (**Figure 3F**) were annotated into 186, 107, 300, 376, 448, and 45 GO items, respectively. Among these, antioxidant and immune-related GO terms mainly included immune response (GO: 0006955), immunoglobulin production (GO: 0002377), positive regulation of the innate immune response (GO: 0045089), hemoglobin complex (GO: 0005833), and antigen binding (GO: 0003823).

#### 3.2.4 KEGG enrichment analysis of differentially expressed genes

KEGG enrichment analysis revealed that the DEGs in the head kidney of rainbow trout among all diet groups were enriched in 191 specific KEGG metabolic pathways. The 29 significant enriched KEGG pathways included metabolism, organismal systems, genetic information processing, cellular processes, and environmental information processing, such as transport and catabolism, signaling molecules and interaction, signal transduction, translation, folding, sorting and degradation, immune system, lipid metabolism, energy metabolism, amino acid metabolism, carbohydrate metabolism, and endocrine system (**Table 8**).

**Table 8:** Significantly altered pathways according to KEGG analysis of the genes whose expression significantly changed in the head kidney of *O. mykiss* fed diets with different sources of astaxanthin.

Pathway	ID	DEGs number					
		NA vs HPA	NA vs PRA	NA vs SA	HPA vs PRA	HPA vs SA	PRA vs SA
Transport and catabolism	ko04144	1	1	2	1		
Eukaryotes	ko04510		1	2	1	1	
Cell growth and death	ko04210	1		3	1		
Signaling molecules and interaction	ko04060	3	1	3	2	3	
Signal transduction	ko04151	2	1	12	8	7	
Membrane transport	ko02010				1		
Translation	ko03008			4	2	3	
Transcription	ko02010				1		
Replication and repair	ko03460		1				
Folding, sorting, and degradation	ko03050	1		6	3	3	
Xenobiotics biodegradation and metabolism	ko00980		1	1		1	
Nucleotide metabolism	ko00230				2	2	1
Metabolism of other amino acids	ko00480			1		1	
Metabolism of cofactors and vitamins	ko00830		1	1	1		
Lipid metabolism	ko01040		3	1	1	1	
Global and overview maps	ko01212		1	1		1	
Energy metabolism	ko04726				2	4	
Carbohydrate metabolism	ko00040		1	2	2	1	1
Biosynthesis of other secondary metabolites	ko00521			1		1	1
Amino acid metabolism	ko00220			3	1	3	
Sensory system	ko04742				2	2	
Nervous system	ko00480				2	4	
Immune system	ko04650	1	2	6	3	3	
Excretory system	ko04961			1			
Endocrine system	ko04925		1	1	2	2	
Digestive system	ko04971			3	1	1	
Development and regeneration	ko04360		1	1	1	1	
Circulatory system	ko04270			1		1	
Aging	ko04211			1			

NA, no astaxanthin; HPA, Haematococcus pluvialis astaxanthin; PRA, Phaffia rhodozyma astaxanthin; SA, synthetic astaxanthin.

### 3.3 Validation of transcriptomic sequencing by quantitative PCR analysis

**Figure 4:**
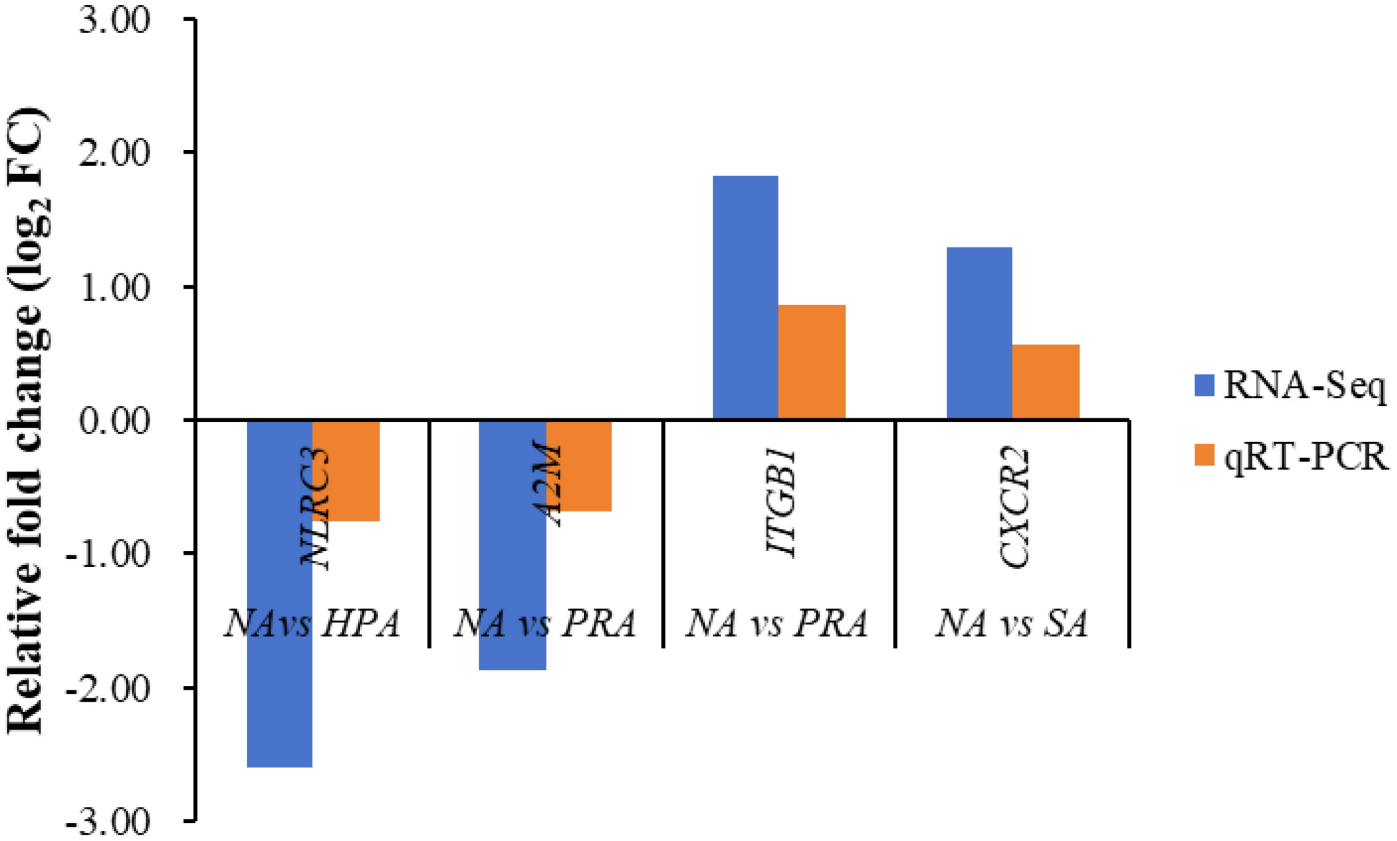
Validation results of RNA-seq profiles by qPCR. The expression of the following proteins was detected by RNA-seq and real-time qPCR. NLRC3, nod like receptor C3; A2M, alpha-2-macroglobulin; ITGB1, integrin beta-1; CXCR2, CXC chemokine receptor type 2.

**Figure 5:**
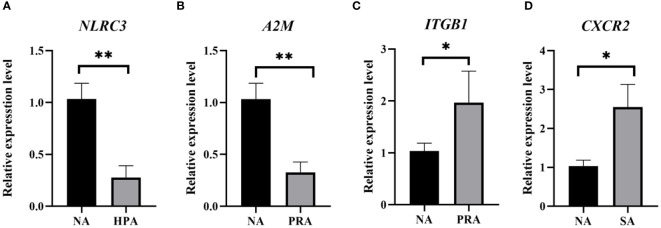
Validation of gene expression patterns in the head kidney of *O. mykiss* by qPCR among different diet groups. The significant differences between the two groups are represented by “*” (*P* < 0.05) and “**” (*P* < 0.01). NA: no astaxanthin; HPA: *Haematococcus pluvialis* astaxanthin; PRA, *Phaffia rhodozyma* astaxanthin; SA, synthetic astaxanthin. **(A)** *NLRC3*, nod like receptor C3; **(B)** *A2M*, alpha-2-macroglobulin; **(C)** *ITGB1*, integrin beta-1; **(D)** *CXCR2*, CXC chemokine receptor type 2.

To validate the expression levels of the DEGs obtained from RNA-Seq data, four DEGs were selected for qRT-PCR analysis using the same RNA samples and the differences of fold changes were compared between RNA-Seq and qRT-PCR. The fold changes from qRT-PCR were consistent with the results from RNA-Seq data, indicating the reliability and accuracy of transcriptome data and quantified gene expression (**Figure 4**). Of the four DEGs selected for qRT-PCR, the expression level of nod like receptor C3 (*NLRC3*) was significantly down-regulated in the HPA group compared with the NA group (**Figure 5A**). The expression level of alpha-2-macroglobulin (*A2M*) was significantly down-regulated (**Figure 5B**), while the expression level of integrin beta-1 (*ITGB1*) was significantly up-regulated in the PRA group compared with the NA group (**Figure 5C**). The expression level of CXC chemokine receptor type 2 (*CXCR2*) was significantly up-regulated in the SA group compared with the NA group (**Figure 5D**).

## 4 Discussion

In this study, OPLS-DA revealed differences in the serum metabolite profiles between the NA and the HPA groups, the NA and the PRA groups, the NA and SA groups, and the HPA and the PRA and the SA groups which indicated that dietary supplementation with astaxanthin had significant effects on the physiological metabolism of commercial-sized rainbow trout. The differential metabolites among the different diet groups were enriched in glycerophospholipid metabolism, linoleic acid metabolism, alpha-linolenic acid metabolism, biosynthesis of unsaturated fatty acids, arginine and proline metabolism, glycine, serine and threonine metabolism and other pathways related to lipid and amino acid metabolism.

Glycerophospholipids are important components of biofilms and play important roles in ensuring the normal operation of membrane-binding proteins, ion channels, and receptors (40). Under the stress condition, the change in cell membrane composition and fluidity is associated with the immune response (41). Previous metabolomics analysis showed that largemouth bass (*Micropterus salmoides*) fed with oxidized fish oil reduced phospholipid unsaturation, which may impair the normal function of cells by affecting the function of receptors in the membrane. This may be one of the causes of increased oxidative stress and decreased immunity in fish (42). In this study, the serum metabolites, such as phosphatidylcholine (PC) and phosphatidyl inositol (PI), which contain multiple double bond structures involved in glycerophospholipid metabolism, were significantly up-regulated in the astaxanthin-supplemented diet groups, resulting in an increase in the unsaturation of phospholipids, thus contributing to the normal operation of receptors in the cell membrane and thus maintaining the normal function of cells.

As one of the important components of the cell membrane, phosphatidylcholine (PC) is a precursor of signaling molecules (43), and plays an important role in the regulation of cell membrane fluidity, permeability, and other functions (44). It can regulate serum lipid levels (such as reducing cholesterol levels), strengthen immunity, and promote growth (45). In this study, serum phosphatidylcholine involved in linoleic acid metabolism and α-linolenic acid metabolism was significantly up-regulated in the HPA, PRA, and SA groups compared with the NA group. These results indicate that the dietary supplementation of astaxanthin is conducive to the biosynthesis of linoleic acid and α-linolenic acid. In addition to reducing blood lipids and participating in the phospholipid composition of cell membranes, linoleic acid can also affect the production of intestinal microbiota and inflammatory mediators (46). Similarly, n-3 poly-unsaturated fatty acids (n-3 PUFAs) such as eicosapentaenoic acid (EPA) and docosahexaenoic acid (DHA) are produced by the extension of the carbon chain under the action of fatty acid desaturase, which has anti-inflammatory effects and plays a key role in regulating body homeostasis (47). This study suggested that dietary supplementation with astaxanthin was beneficial to the accumulation of fatty acids in commercial-sized rainbow trout, which may be conducive to enhancing the polyunsaturated fatty acid content, increasing the nutritional value, and improving the antioxidant capacity.

The head kidney is an important immune and hematopoietic organ in teleost fish (48–50) and plays an important physiological role in the initial stage of the immune response (51). In this study, antioxidant and immune-related differentially expressed genes (DEGs), such as interleukin-2 receptor subunit beta (*IL2RB*) and the glycine receptor subunit alpha Z1 (*GLRA1*) were detected between the NA and HPA groups; alpha-2-macroglobulin (*A2M*) and integrin beta-1 (*ITGB1*), between the NA and PRA groups, and CXC chemokine receptor type 2 (*CXCR2*), CCAAT/enhancer-binding protein beta (*CEBPB*), dual specificity protein phosphatase 1 (*DUSP1*), growth arrest and DNA damage-inducible protein GADD45 alpha (*GADD45A*), ornithine decarboxylase 1 (*ODC1A*), and diamine acetyltransferase 1 (*SAT1*), between the NA and SA groups. These DEGs were mainly involved in signaling pathways related to immunity, anti-inflammation, and antioxidants, including cell adhesion molecules (*CAMs*), ubiquitin-mediated proteolysis, *FoxO* signaling pathway, phagosome, complement and coagulation cascades, natural killer cell-mediated cytotoxicity, RIG-I-like receptor signaling pathway, NF-kappa B signaling pathway, TNF signaling pathway, arginine and proline metabolism, glutathione metabolism, etc.

Cell adhesion molecules (*CAMs*) can promote the migration of immune cells during immune responses (52). The integrin family is one of the categories of cell adhesion molecules, and it is a heterodimeric transmembrane glycoprotein that includes α and β subunits and plays important roles in cell migration, differentiation, and survival (53). Previous studies have shown that integrin subunits were cellular receptors for white spot syndrome virus infection in Chinese shrimp (*Fenneropenaeus chinensis*) (54, 55) and Kuruma prawn (*Marsupenaeus japonicas*) (56). The antibacterial activity of integrins has been reported in Chinese mitten crab (*Eriocheir sinensis*) (53). Additionally, integrins are involved in precursor activation, phagocytosis, and immunomodulatory antioxidant systems in Pacific white shrimp (*Litopenaeus vannamei*) (57). In this study, the head kidney DEGs between the NA and PRA groups were enriched in integrin binding, integrin-mediated signaling pathway, integrin complex, and other related pathways. Significantly different signaling pathways, CAMs, and related DEGs (such as Integrin beta-1 (*ITGB1*) and Claudin-4 (*CLDN4*)) were found between the NA and PRA groups and between the HPA and SA groups. These results suggested that the addition of astaxanthin from different sources may enhance the mutual recognition and adhesion between cells and extracellular mediators by enhancing integrins, thus improving the immunity of fish.

The ubiquitin proteolytic system plays important roles in regulations of the cell cycle, and immune and inflammatory responses, as well as in the control of signal transduction pathways, development, and differentiation (58). In fish, studies on ubiquitin-mediated proteolysis have focused mainly on viral infection (59). The expression of ubiquitin-related proteins in rainbow trout is disrupted after bacterial infection (60), and ubiquitin degradation can promote MHC molecule synthesis, the endoplasmic reticulum pathway, antigen processing and delivery, etc., to assist the body’s immune system clearing exogenous antigens. In this study, the E3 ubiquitin/ISG15 ligase TRIM25 (*TRIM25*) was significantly up-regulated in the PRA group compared with the NA group. This result suggested that the addition of *P. rhodozyma* astaxanthin to the diet could maintain the normal translation of the protein to a certain extent, thus enhancing the immune response.

Chemokines are cytokines that mediate the migration of immune cells. When the body is injured or infected, chemokines will chemoattract immune-related cells to the site of injury or infection and bind to G protein-coupled receptors on target cells, leading tochemotaxis (61). In response to pathogenic exposure, chemokines not only promote leukocyte mobilization but also regulate immune responses and the differentiation of recruited cells to orchestrate the first steps of both innate and adaptive immunity (62, 63). Previous studies have shown that the expression of *CXCR2* increased in the kidneys, spleen, and liver of large yellow croaker (*Larimichthys crocea*) after challenge with *Vibrio anguillarum*; likely, high expression of *CXCR2* was also detected in the kidneys and spleen of rock bream (*Oplegnathus fasciatus*) (64). These studies suggested that *CXCR2* was very important in resisting bacterial infection (65). A study by Hu et al. (2020) showed that *CXCR2* was highly expressed in the spleen, intestine, liver, and head kidney of tilapia after infection with *Streptococcus agalactiae* and *Aeromonas hydrophila*, which indicates that *CXCR2* plays a role in the immune response (62). In this study, the expression level of the head kidney gene *CXCR2*, which is involved in the chemokine signaling pathway, was significantly up-regulated in the SA group compared to the NA group. This result indicated that the addition of synthetic astaxanthin to the diet was beneficial in enhancing the immune function of rainbow trout, which may be related to the gene expression of cytokines, chemotaxis, and several immunity-related signaling molecules (66). These results will provide a basis for further research on the function of *CXCR2*.

The *FoxO* signaling pathway is related to a variety of biological processes, including cell metabolism, immunity, and antioxidant stress (67). In terms of oxidative stress, the *FoxO* signaling pathway can drive the expression of genes involved in fighting oxidative stress and accelerating cell cycle circulation, thereby protecting cell function (68) Gao et al. (2019) reported that *FoxO* plays an important role in the immune response to bacterial infection in Channel catfish (*Ictalurus punctatus*) (69). In this study, the head kidney gene, growth arrest, and DNA damage-inducible protein GADD45 alpha (*GADD45A*), which is involved in the *FoxO* signaling pathway, were significantly up-regulated in the SA group compared with the NA group, which may represent the host’s early defense against bacterial infection.

Phagocytosis is a central mechanism of tissue remodeling, inflammation, and defense against infection, and it is the process by which cells transport invading pathogens into cells for elimination (70). In addition to providing cells with specific immune functions, phagocytes are also an important part of the non-specific defense system and play an important role in all stages of foreign pathogen invasion in fish (71, 72). A study by Song et al. (2021) showed that the phagosome pathway was significantly enriched in the head kidney of red sea bream (*Pagrus major*) after infection with *Vibrio anguillarum* (9). Transcriptomics analysis results showed that phagosome pathways related to immunity were also enriched in rainbow trout after heat stress (73). In this study, head kidney genes, such as integrin beta-1 (*ITGB1*) and immunoglobulin heavy variable 3-43 (*IGHV3-43*), which are involved in phagosome pathways, were significantly up-regulated in the PRA group compared with the NA and HPA groups. These results indicated that the addition of astaxanthin could improve the body’s ability to fight inflammation and defend against infection, which may be related to the strong antioxidant and immune functions of astaxanthin.

The complement system, the earliest evolved immune response system, exists in the serum and can mediate a variety of functions (including phagocytosis, cytolysis, inflammation, dissolution of immune complexes, clearance of apoptotic cells, and promotion of humoral immune response), mainly by regulating antigens to assist antigen phagocytosis, cell lysis, and chemotaxis. The complement system not only plays an important role in innate immunity but also regulates the level of specific immune responses by assisting antigen phagocytosis (74–77). After invasion, complement binds to the surface of the pathogen, inducing phagocytes to recognize and engulf the pathogen (76, 78). In this study, the expression of head kidney genes involved in the complement and coagulation cascades, such as alpha-2-macroglobulin (*A2M*) and plasminogen activator inhibitor 1 (*SERPINE1*), was up-regulated in the PRA and SA groups compared to the NA group. *A2M* is a member of the alpha-macroglobulin family (α-macroglobulin, αMs), a class of macromolecular glycoproteins present in a variety of animal plasma, and has been shown to regulate host cell apoptosis and cancer cell metabolism, enhance prothrombin activation and thrombin potential, mediate T-cell proliferation, and induce macrophage proliferation and activation (79, 80). It has been reported that the *A2M* and complement components (C3 and C4) of *L. vannamei* are highly conserved during evolution and play a certain role in bacterial infection (81, 82). Variable splicing of the *A2M* gene also plays a role in immune diseases in carp and shrimp (83, 84). In pearl oysters (*Pinctada fucata*), *A2M* significantly reduced the *in vivo* phagocytosis of *Vibrio alginolyticus* by blood cells (85). In addition, in the swimming crab (*Portunus trituberculatus*), *PtA2Ms* may play a role in host defense by regulating phagocytosis and the expression of antimicrobial peptide genes (79). In general, these genes play an important role in the initial stage of the immune response, promoting innate immunity development, indicating that the complement and coagulation cascade systems are activated and work synergistically to eliminate bacteria, which is conducive to enhancing the immunity of rainbow trout.

In this study, the arginine and proline metabolism pathway exists in both metabolomics analysis and transcriptomic analysis results. Arginine and proline are two important fatty acid amides that play important roles in osmotic stress. Arginine can produce nitric oxide (NO), and participate in immune function regulation in fish (15). Some studies have shown that arginine affects the growth performance, immunity, and nutrient metabolism of fish (86, 87), and the metabolism of arginine and proline is closely related to the progression of oxidative stress (88, 89). Proline plays important roles in protein synthesis and structure, metabolism and nutrition, as well as wound healing, antioxidative reactions, and immune responses (90). In this study, the expression levels of diamine acetyltransferase 1 (*SAT1*) and ornithine decarboxylase 1 (*ODC1A*) which are involved in the proline and arginine metabolism pathways, were significantly up-regulated in the SA group. This result suggested that dietary supplementation with synthetic astaxanthin may contribute to the maintenance of osmotic pressure and resistance to oxidative stress.

In addition, both the transcriptome and metabolome pathway enrichment results showed the presence of lipid metabolism and amino acid metabolism pathways. Further analysis of the data reveled that PRA, compared to NA, screened out the gene *HSD17B12A* (very-long-chain 3-oxoacyl-CoA reductase-A), which is related to lipid metabolism and specifically involved in fatty acid metabolism and biosynthesis of unsaturated fatty acids. Additionally, the genes *PLPP1* (phospholipid phosphatase 1), *ARG2* (arginase-2 and mitochondrial), and *ODC1-A* (ornithine decarboxylase 1) were screened in both the SA and NA groups. *PLPP1* is primarily involved in glycerophospholipid metabolism, *ARG2* is involved in the biosynthesis of amino acids and arginine and proline metabolism, and *ODC1-A* is mainly involved in arginine and proline metabolism and glutathione metabolism.

*PLPP1* is a multifunctional phospholipase that can catalyze the hydrolysis of various fatty acid phospholipids. Studies have shown that cholic acid has a hepatoprotective effect on CCL4-induced acute liver injury in mice by regulating the lipid metabolism pathway (especially the metabolism of glycerophospholipids, arachidonic acid, and linoleic acid) and altering the expression of *Ptgr1* and *PLPP1* genes (91). *ARG2* is a mitochondrial enzyme involved in arginine metabolism that promotes the resolution of macrophage inflammation and is a target of microRNA-155 (*miR-155*). Mechanistically, the catalytic activity and presence of ARG2 in mitochondria are critical for oxidative phosphorylation. Moreover, *ARG2* is essential for IL-10-mediated downregulation of the inflammatory mediators succinate, hypoxia-inducible factor 1α (*HIF-1α*), and IL-1β *in vitro* (92, 93). These results suggest that the addition of *P. rhodozyme* astaxanthin and synthetic astaxanthin can alter the body’s antioxidant and immune responses by up-regulating the expression of genes related to amino acid and lipid metabolism.

## 5 Conclusion

This study showed that dietary supplementation with astaxanthin was conducive to the biosynthesis of linoleic acid, α-linolenic acid, arachidonic acid and glycerophospholipids, significantly increased the content of astaxanthin in the serum of rainbow trout, and was conducive to improving the health status; the contents of phosphatidylcholine and phosphatidylinositol were significantly increased, which was helpful to regulate the stability of cell membrane function of rainbow trout. Dietary supplementation with astaxanthin could enhance the antioxidant and immune functions of rainbow trout by affecting the expression of integrin beta-1 (*ITGB1*), CXC chemokine receptor type 2 (*CXCR2*), and ornithine decarboxylase 1 (*ODC1A*) and regulating the *FoxO* signaling pathway, phagosome, complement and coagulation cascade, and arginine and proline metabolism pathways. These results provide some information for further understanding of the molecular mechanism related to astaxanthin regulation of antioxidant and immune functions in rainbow trout and provide a reference for the healthy and sustainable development of rainbow trout aquaculture.

## Data availability statement

The datasets presented in this article are not readily available due to the deletion of the raw data by a third-party. Requests to access the datasets should be directed to the corresponding author.

## Ethics statement

The animal study was approved by the Animal Ethics Committee of Dali University. The study was conducted in accordance with the local legislation and institutional requirements.

## Author contributions

LC: Conceptualization, Investigation, Methodology, Writing – original draft. LW: Formal analysis, Investigation, Methodology, Writing – original draft. YL: Formal Analysis, Investigation, Writing – review & editing. XW: Conceptualization, Funding acquisition, Methodology, Supervision, Writing – review & editing. XL: Conceptualization, Funding acquisition, Methodology, Supervision, Writing – review & editing.

## References

[1] Rebolé S Velasco S Rodriguez ML Treviño J Alzueta C Tejedor JL. Nutrient content in the muscle and skin of fillets from farmed rainbow trout (Oncorhynchus mykiss). Food Chem. (2015) 174:614–20. doi: 10.1016/j.foodchem.2014.11.072 25529727

[2] Bondad-Reantaso MG Subasinghe RP Arthur JR Ogawa K Chinabut S Adlard R. Disease and health management in Asian aquaculture. Vet Parasitol. (2005) 132:249–72. doi: 10.1016/j.vetpar.2005.07.005 16099592

[3] Carbone D Faggio C. Importance of prebiotics in aquaculture as immunostimulants. Effects on immune system of Sparus aurata and Dicentrarchus labrax. Fish Shellfish Immun. (2016) 54:172–8. doi: 10.1016/j.fsi.2016.04.011 27074444

[4] Round JL Stentiford GD Sritunyalucksana K Flegel TW Williams BAP Withyachumnarnkul B. New paradigms to help solve the global aquaculture disease crisis. PloS Pathog. (2017) 13:e1006160. doi: 10.1371/journal.ppat.1006160 28152043 PMC5289612

[5] Raza SHA Naqvi SRZ Abdelnour SA Schreurs N Mohammedsaleh ZM Khan I. Beneficial effects and health benefits of astaxanthin molecules on animal production: A review. Res Vet Sci. (2021) 138:69–78. doi: 10.1016/j.rvsc.2021.05.023 34111716

[6] Li M Wu W Zhou P Xie F Zhou Q Mai K. Comparison effect of dietary astaxanthin and Haematococcus pluvialis on growth performance, antioxidant status and immune response of large yellow croaker Pseudosciaena crocea. Aquaculture. (2014) 434:227–32. doi: 10.1016/j.aquaculture.2014.08.022

[7] Cao Y Yang L Qiao X Xue C Xu J. Dietary astaxanthin: an excellent carotenoid with multiple health benefits. Crit Rev Food Sci. (2021) 63:3019–45. doi: 10.1080/10408398.2021.1983766 34581210

[8] Chang MX Xiong F. Astaxanthin and its effects in inflammatory responses and inflammation-associated diseases: recent advances and future directions. Molecules. (2020) 25:5342. doi: 10.3390/molecules25225342 33207669 PMC7696511

[9] Song XL Wang L Li XQ Chen ZZ Liang GY Leng XJ. Dietary astaxanthin improved the body pigmentation and antioxidant function, but not the growth of discus fish (Symphysodon spp). Aquac Res. (2017) 48:1359–67. doi: 10.1111/are.13200

[10] Wu XG Zhao L Long XW Liu JG Su F Cheng YX. Effects of dietary supplementation of Haematococcus pluvialis powder on gonadal development, coloration and antioxidant capacity of adult male Chinese mitten crab (Eriocheir sinensis). Aquac Res. (2017) 48:5214–23. doi: 10.1111/are.13333

[11] Bjerkeng B Peisker M von Schwartzenberg K Ytrestøyl T Åsgård T. Digestibility and muscle retention of astaxanthin in Atlantic salmon, Salmo salar, fed diets with the red yeast Phaffia rhodozyma in comparison with synthetic formulated astaxanthin. Aquaculture. (2007) 269:476–89. doi: 10.1016/j.aquaculture.2007.04.070

[12] Ishibashi T. Manufacture production of carotenoid from Paracoccus bacterium. Seibutsu-kogaku kaishi. (2015) 93:388–90.

[13] Roncarati A Sirri F Felici A Stocchi L Melotti P Meluzzi A. Effects of dietary supplementation with krill meal on pigmentation and quality of flesh of rainbow trout (Oncorhynchus mykiss). Ital J Anim Sci. (2016) 10:139–45. doi: 10.4081/ijas.2011.e27

[14] European, Food, Safety Authority. Scientific Opinion of the Panel on Biological Hazards (BIOHAZ) - Monitoring of verotoxigenic Escherichia coli (VTEC) and identification of human pathogenic VTEC types. Efsa J. 5:579 (2007).

[15] Sheikhzadeh N Tayefi-Nasrabadi H Oushani AK Enferadi MH. Effects of Haematococcus pluvialis supplementation on antioxidant system and metabolism in rainbow trout (Oncorhynchus mykiss). Fish Physiol Biochem. (2012) 38:413–9. doi: 10.1007/s10695-011-9519-7 21695482

[16] Elia AC Prearo M Dörr AJM Pacini N Magara G Brizio P. Effects of astaxanthin and canthaxanthin on oxidative stress biomarkers in rainbow trout. J Toxicol Env Heal A. (2019) 82:760–8. doi: 10.1080/15287394.2019.1648346 31370749

[17] Ju ZY Deng DF Dominy WG Forster IP. Pigmentation of pacific white shrimp, Litopenaeus vannamei, by dietary astaxanthin extracted from Haematococcus pluvialis. J World Aquacul Soc. (2011) 42:633–44. doi: 10.1111/j.1749-7345.2011.00511.x

[18] Liu XJ Luo QX Rakariyatham K Cao Y Goulette T Liu X. Antioxidation and anti-ageing activities of different stereoisomeric astaxanthin in *vitro* and in vivo. J Funct Foods. (2016) 25:50–61. doi: 10.1016/j.jff.2016.05.009

[19] Liu XJ Luo QX Cao Y Goulette T Liu X Xiao H. Mechanism of different stereoisomeric astaxanthin in resistance to oxidative stress in Caenorhabditis elegans. J Food Sci. (2016) 81:H2280–7. doi: 10.1111/1750-3841.13417 27527357

[20] Rao AR Sindhuja HN Dharmesh SM Sankar KU Sarada R Ravishankar GA. Effective inhibition of skin cancer, tyrosinase, and antioxidative properties by astaxanthin and astaxanthin esters from the green alga Haematococcus pluvialis. J Agric Food Chem. (2013) 61:3842–51. doi: 10.1021/jf304609j 23473626

[21] Choi J Rahman MM Le SY Chang KH Lee SM. Effects of dietary inclusion of fermented soybean meal with Phaffia rhodozyma on growth, muscle pigmentation, and antioxidant activity of juvenile rainbow trout (Oncorhynchus mykiss). Turk J Fish Aquat Sci. (2016) 16:91–101. doi: 10.4194/1303-2712-v16_1_10

[22] Nakano T Kanmuri T Sato M Takeuchi M. Effect of astaxanthin rich red yeast (Phaffia rhodozyma) on oxidative stress in rainbow trout. Bba-Bioenergetics. (1999) 1426:119–25. doi: 10.1016/S0304-4165(98)00145-7 9878705

[23] Amar E Kiron V Satoh S Watanabe T. Enhancement of innate immunity in rainbow trout (Oncorhynchus mykiss Walbaum) associated with dietary intake of carotenoids from natural products. Fish Shellfish Immun. (2004) 16:527–37. doi: 10.1016/j.fsi.2003.09.004 15123294

[24] Christiansen R Torrissen OJ. Growth and survival of Atlantic salmon, Salmo salar L. fed different dietary levels of astaxanthin. Juveniles Aquacult Nutr. (1996) 2:55–62. doi: 10.1111/j.1365-2095.1996.tb00008.x

[25] Zhang JJ Li XQ Leng XJ Zhang CL Han ZY Zhang FG. Effects of dietary astaxanthins on pigmentation of flesh and tissue antioxidation of rainbow trout (Oncorhynchus mykiss). Aquacul Int. (2013) 21:579–89. doi: 10.1007/s10499-012-9590-9

[26] Valenzuela-Muñoz V Valenzuela-Miranda D Gallardo-Escárate C. Comparative analysis of long non-coding RNAs in Atlantic and Coho salmon reveals divergent transcriptome responses associated with immunity and tissue repair during sea lice infestation. Dev Com Immunol. (2018) 87:36–50. doi: 10.1016/j.dci.2018.05.016 29803715

[27] Saghatelian A Cravatt BF. Global strategies to integrate the proteome and metabolome. Curr Opin Chem Biol. (2005) 9:62–8. doi: 10.1016/j.cbpa.2004.12.004 15701455

[28] Utpott M Rodrigues E Rios AD Mercali GD Flo SH. Metabolomics: An analytical technique for food processing evaluation. Food Chem. (2021) 366:130685. doi: 10.1016/j.foodchem.2021.130685 34333182

[29] Konga TT Lin SM Ren X Li SK Gong Y. Transcriptome and metabolome integration analysis of mud crab Scylla paramamosain challenged to Vibrio parahaemolyticus infection. Fish Shellfish Immun. (2020) 103:430–7. doi: 10.1016/j.fsi.2020.05.069 32473364

[30] Wilhelm BT Landry JR. RNA-Seq-quantitative measurement of expression through massively parallel RNA-sequencing. Methods. (2009) 48:249–57. doi: 10.1016/j.ymeth.2009.03.016 19336255

[31] Alfaro AC Young T. Showcasing metabolomic applications in aquaculture: a review. Rev Aquacult. (2018) 10:135–52. doi: 10.1111/raq.12152

[32] Ma QQ Chen Q Shen ZH Li DL Han T Qin JG. The metabolomics responses of Chinese mitten-hand crab (Eriocheir sinensis) to different dietary oils. Aquaculture. (2017) 479:188–99. doi: 10.1016/j.aquaculture.2017.05.032

[33] Patti GJ Yanes O Siuzdak G. Metabolomics: the apogee of the omics trilogy. Nat Rev Mol Cell Bio. (2012) 13:263–9. doi: 10.1038/nrm3314 PMC3682684 22436749

[34] Roques S Deborde C Richard N Skiba-Cassy S Moing A Fauconneau B. Metabolomics and fish nutrition: a review in the context of sustainable feed development. Rev aquacult. (2018) 12:261–82. doi: 10.1111/raq.12316

[35] Yang H Gao X Li X Zhang HH Chen N Zhang YY. Comparative transcriptome analysis of red swamp crayfish (Procambarus clarkia) hepatopancreas in response to WSSV and Aeromonas hydrophila infection. Fish Shellfish Immun. (2018) 83:397–405. doi: 10.1016/j.fsi.2018.09.051 30244087

[36] Ning X Sun L. Gene network analysis reveals a core set of genes involved in the immune response of Japanese flounder (Paralichthys olivaceus) against Vibrio Anguillarum infection. Fish Shellfish Immun. (2020) 98:800–9. doi: 10.1016/j.fsi.2019.11.033 31743762

[37] Chu Q Gao YH Xu GL Wu CW Xu TJ. Transcriptome comparative analysis revealed poly(I:C) activated RIG-I/MDA5-mediated signaling pathway in miiuy croaker. Fish Shellfish Immun. (2015) 47:168–74. doi: 10.1016/j.fsi.2015.08.032 26334792

[38] AOAC, Association of Official Analytical Chemists. Official methods of analysis. Gait Hersburg Maryland, USA: Association of Official Analytical Chemists (2000).

[39] Folch J Lees M Stanley G. A simple method for the isolation and purification of total lipides from animal tissues. J Biol Chem. (1957) 226:497–509. doi: 10.1016/S0021-9258(18)64849-5 13428781

[40] Fokina NN Ruokolainen TR Bakhmet IN Nemova NN. Role of lipids in adaptation of mussels Mytilus edulis L. of the White Sea to rapid changes in temperature. Dokl Biochem Biophys. (2014) 457:155–7. doi: 10.1134/S1607672914040103 25172340

[41] Arts MT Kohler CC. Health and condition in fish: the influence of lipids on membrane competency and immune response. Lipids in Aquatic Ecosystems. (2009), 237–56. doi: 10.1007/978-0-387-89366-2_10

[42] Xie SW Yin P Tian LX Liu YJ Niu J. Lipid metabolism and plasma metabolomics of juvenile largemouth bass Micropterus salmoides were affected by dietary oxidized fish oil. Aquaculture. (2020) 522:735158. doi: 10.1016/j.aquaculture.2020.735158

[43] Cahu CL Infante JZ Barbosa V. Effect of dietary phospholipid level and phospholipid: neutral lipid value on the development of sea bass (Dicentrarchus labrax) larvae fed a compound diet. Brit J Nutr. (2003) 90:21–8. doi: 10.1079/BJN2003880 12844371

[44] Robinson BS Yao ZM Baisted DJ Vance DE. Lyso-phosphatidylcholine metabolism and lipoprotein secretion bycultured rat hepatocytes deficient in choline. Biochem J. (1989) 260:207–14. doi: 10.1042/bj2600207 PMC1138647 2775183

[45] Krogdahl Å Hansen AKG Kortner TM Björkhem I Krasnov A Berge GM. Choline and phosphatidylcholine, but not methionine, cysteine, taurine and taurocholate, eliminate excessive gut mucosal lipid accumulation in Atlantic salmon (Salmo salar L). Aquaculture. (2020) 528:735552. doi: 10.1016/j.aquaculture.2020.735552

[46] Lou MS Yu H Shen SR. Association of dietary linoleic acid with gut microbiome and chronic metabolic diseases. J Mod Med Health. (2013) 39:3611–9. doi: 10.3969/j.issn.1009-5519.2023.21.003

[47] Saini RK Keum YS. Omega-3 and omega-6 polyunsaturated fatty acids: Dietary sources, metabolism, and significance: A review. Life Sci. (2018) 203:255–67. doi: 10.1016/j.lfs.2018.04.049 29715470

[48] Zhong AH Dai XX. Comparative transcriptome analysis of the head kidney and trunk kidney in adult yellow catfish (Pelteobagrus fulvidraco). Oceanologia Limnologia Sin. (2021) 52:1486–95. doi: 10.11693/hyhz20210500110

[49] Jing HY Zhang QR Li S Gao XJ. Pb exposure triggers MAPK-dependent inflammation by activating oxidative stress and miRNA-155 expression in carp head kidney. Fish Shellfish Immun. (2020) 106:219–27. doi: 10.1016/j.fsi.2020.08.015 32781208

[50] Song LF Dong XZ Hu GB. Transcriptome analysis of red sea bream (Pagrus major) head kidney and spleen infected by Vibrio Anguillarum. Aquacult Rep. (2021) 21:100789. doi: 10.1016/j.aqrep.2021.100789

[51] Castro R Coll J Blanco MM Rodriguez-Bertos A. Spleen and head kidney differential gene expression patterns in trout infected with Lactococcus garvieae correlate with spleen granulomas. Vet Res. (2019) 50:32. doi: 10.1186/s13567-019-0649-8 31046823 PMC6498643

[52] Ge H Lin KB Zhou C Lin Q Zhang ZP Wu JS. A multi-omic analysis of orange-spotted grouper larvae infected with nervous necrosis virus identifies increased adhesion molecules and collagen synthesis in the persistent state. Fish Shellfish Immun. (2020) 98:595–604. doi: 10.1016/j.fsi.2020.01.056 32004615

[53] Huang Y Zhao LL Feng JL Zhu HX Huang X Ren Q. A novel integrin function in innate immunity from Chinese mitten crab (Eriocheir sinensis). Dev Comp Immunol. (2015) 52:155–65. doi: 10.1016/j.dci.2015.05.005 26004499

[54] Escobedo-Bonilla CM Alday- Sanz V Wille M Sorgeloos P Pensaert MB Nauwynck HJ. A review on the morphology, molecular characterization, morphogenesis and pathogenesis of white spot syndrome virus. J Fish Dis. (2008) 31:1–18. doi: 10.1111/j.1365-2761.2007.00877.x 18086030

[55] Tang X Wang X Zhan W. An integrin β subunit of Chinese shrimp Fenneropenaeus chinensis involved in WSSV infection. Aquaculture. (2012) 368:1–9. doi: 10.1016/j.aquaculture.2012.08.004

[56] Li DF Zhang MC Yang HJ Zhu YB Xu X. beta-integrin mediates WSSV infection. Virology. (2007) 368:122–32. doi: 10.1016/j.virol.2007.06.027 17655902

[57] Lin YC Chen JC Chen YY Liu CH Cheng W Hsu CH. Characterization of white shrimp Litopenaeus vannamei integrin beta and its role in immunomodulation by dsRNA-mediated gene silencing. Dev Comp Immunol. (2013) 40:167–79. doi: 10.1016/j.dci.2013.01.001 23376419

[58] Tsukamoto S Yokosawa H. Natural products inhibiting the ubiquitin-proteasome proteolytic pathway, a target for drug development. Curr Med Chem. (2006) 13:745–54. doi: 10.2174/092986706776055571 16611064

[59] Huang RQ Zhang J Zhu GH He JG Xie JF. The core ubiquitin system of mandarin fish, Siniperca chuatsi, can be utilized by infectious spleen and kidney necrosis virus. Fish Shellfish Immun. (2017) 70:293–301. doi: 10.1016/j.fsi.2017.09.017 28889013

[60] Rivas-Aravena A Fuentes-Valenzuela M Escobar-Aguirre S Gallardo-Escarate C Molina A Valdés JA. Transcriptomic response of rainbow trout (Oncorhynchus mykiss) skeletal muscle to Flavobacterium psychrophilum. Comp Biochem Phys D. (2019) 31:100596. doi: 10.1016/j.cbd.2019.100596 31174158

[61] Hu QM Ao QW Zhu JJ. Response of chemokine receptors CXCR2 and integrin β2 after Streptococcus agalactiae and Aeromonas hydrophila challenge in GIFT strain of Nile tilapia Oreochromis niloticus. Dev Comp Immunol. (2020) 115:103897. doi: 10.1016/j.dci.2020.103897 33132113

[62] Bird S Tafalla C. Teleost chemokines and their receptors. Biology. (2015) 4:756–84. doi: 10.3390/biology4040756 PMC4690017 26569324

[63] Luo K Di J Han PP Zhang SH Xia LH Tian GM. Transcriptome analysis of the critically endangered Dabry's sturgeon (Acipenser dabryanus) head kidney response to Aeromonas hydrophila. Fish Shellfish Immun. (2018) 83:249–61. doi: 10.1016/j.fsi.2018.09.044 30219387

[64] Umasuthan N Wan Q Revathy KS Whang I Noh JK Kim S. Molecular aspects, genomic arrangement and immune responsive mRNA expression profiles of two CXC chemokine receptor homologs (CXCR1 and CXCR2) from rock bream, Oplegnathus fasciatus. Fish Shellfish Immun. (2014) 40:304–18. doi: 10.1016/j.fsi.2014.06.006 24945570

[65] Liu XX Kang LS Liu W Lou B Wu CW Jiang LH. Molecular characterization and expression analysis of the large yellow croaker (Larimichthys crocea) chemokine receptors CXCR2, CXCR3, and CXCR4 after bacterial and poly I:C challenge. Fish Shellfish Immun. (2017) 70:228–39. doi: 10.1016/j.fsi.2017.08.029 28870858

[66] Paneru B Al-Tobasei R Palti Y Wiens GD Salem M. Differential expression of long non-coding RNAs in three genetic lines of rainbow trout in response to infection with Flavobacterium psychrophilum. Sci REP-UK. (2016) 6:36032. doi: 10.1038/srep36032 PMC5081542 27786264

[67] Lees J Hay J Moles MW Michie AM. The discrete roles of individual FOXO transcription factor family members in B-cell Malignancies. Front Immunol. (2023) 14:1179101. doi: 10.3389/fimmu.2023.1179101 37275916 PMC10233034

[68] Gross DN van den Heuvel APJ Birnbaum MJ. The role of FoxO in the regulation of metabolism. Oncogene. (2008) 27:2320–36. doi: 10.1038/onc.2008.25 18391974

[69] Gao L Yuan ZH Zhou T Yang YJ Gao DY Dunham R. FOXO genes in channel catfish and their response after bacterial infection. Dev Comp Immunol. (2019) 97:38–44. doi: 10.1016/j.dci.2019.03.010 30905685

[70] Zhao LJ Tu JG Zhang YL Wang JF Yang L Wang WM. Transcriptomic analysis of the head kidney of Topmouth culter (Culter alburnus) infected with Flavobacterium columnare with an emphasis on phagosome pathway. Fish Shellfish Immun. (2016) 57:413–8. doi: 10.1016/j.fsi.2016.09.001 27601296

[71] Herskovits AA Auerbuch V Portnoy DA. Bacterial ligands generated in a phagosome are targets of the cytosolic innate immune system. PloS Pathog. (2007) 3:e51. doi: 10.1371/journal.ppat.0030051 17397264 PMC1839167

[72] Rieger AM Barreda DR. Antimicrobial mechanisms of fish leukocytes. Dev Comp Immunol. (2011) 35:1238–45. doi: 10.1016/j.dci.2011.03.009 21414350

[73] Huang JQ Li YJ Liu Z Kang YJ Wang JF. Transcriptomic responses to heat stress in rainbow trout Oncorhynchus mykiss head kidney. Fish Shellfish Immunol. (2018) 82:32–40. doi: 10.1016/j.fsi.2018.08.002 30077801

[74] Amara U Rittirsch D Flierl M Bruckner U Klos A Gebhard F. Interaction between the coagulation and complement system. Curr Top complement II. (2008) 632:71–9. doi: 10.1007/978-0-387-78952-1_6 PMC2713875 19025115

[75] Markiewski MM Nilsson B Ekdahl KN Mollnes TE Lambris JD. Complement and coagulation: strangers or partners in crime? Trends Immunol. (2007) 28:184–92. doi: 10.1016/j.it.2007.02.006 17336159

[76] Nakao M Tsujikura M Ichiki S Vo TK Somamoto T. The complement system in teleost fish: progress of post-homolog-hunting researches. Dev Comp Immunol. (2011) 35:1296–308. doi: 10.1016/j.dci.2011.03.003 21414344

[77] Wang LL Zhang H Wang LL Zhang DX Lv Z Liu ZQ. The RNA-seq analysis suggests a potential multi-component complement system in oyster Crassostrea gigas. Dev Comp Immunol. (2017) 76:209–19. doi: 10.1016/j.dci.2017.06.009 28645512

[78] Chen YX Xu KD Li JJ Wang XY Ye YY Qi PZ. Molecular characterization of complement component 3 (C3) in Mytilus coruscus improves our understanding of bivalve complement system. Fish Shellfish Immun. (2018) 76:41–7. doi: 10.1016/j.fsi.2018.02.044 29486351

[79] Ning JH Liu Y Gao FT Song CW Cui ZX. Two alpha-2 macroglobulin from Portunus trituberculatus involved in the prophenoloxidase system, phagocytosis and regulation of antimicrobial peptides. Fish Shellfish Immun. (2019) 89:574–85. doi: 10.1016/j.fsi.2019.04.033 30995541

[80] Pizzo SV. When is a proteinase inhibitor a hormone? The strange tale of α2-macroglobulin. J Nat Sci. (2015) 1:1–5.

[81] Ho PY Cheng CH Cheng WT. Identification and cloning of the alpha2-macroglobulin of giant freshwater prawn Macrobrachium rosenbergii and its expression in relation with the molt stage and bacteria injection. Fish Shellfish Immun. (2009) 26:459–66. doi: 10.1016/j.fsi.2009.01.007 19340942

[82] Lin YC Vaseeharan B Chen JC. Molecular cloning and phylogenetic analysis on α2-macroglobulin (α2-M) of white shrimp Litopenaeus vannamei. Dev Comp Immunol. (2008) 32:317–29. doi: 10.1016/j.dci.2007.07.002 17706773

[83] Ma HM Wang B Zhang JQ Li FH Xiang JH. Multiple forms of alpha-2 macroglobulin in shrimp Fenneropenaeus chinesis and their transcriptional response to WSSV or Vibrio pathogen infection. Dev Comp Immunol. (2010) 34:677–84. doi: 10.1016/j.dci.2010.01.014 20105438

[84] Onara DF Forlenza M Gonzalez SF Rakus KL Pilarczyk A Irnazarow G. Differential transcription of multiple forms of alpha-2-macroglobulin in carp (Cyprinus carpio) infected with parasites. Dev Comp Immunol. (2008) 32:339–47. doi: 10.1016/j.dci.2007.06.007 17662386

[85] Wang ZL Wang B Chen G Lu YS Jian JC Wu ZH. An alpha-2 macroglobulin in the pearl oyster Pinctada fucata: characterization and function in hemocyte phagocy-tosis of Vibrio alginolyticus. Fish Shellfish Immun. (2016) 55:585–94. doi: 10.1016/j.fsi.2016.06.037 27346151

[86] Chen GF Liu Y Jiang J Jiang WD Kuang SY Tang L. Effect of dietary arginine on the immune response and gene expression in head kidney and spleen following infection of Jian carp with Aeromonas hydrophila. Fish Shellfish Immun. (2015) 44:195–202. doi: 10.1016/j.fsi.2015.02.027 25721332

[87] Andersen SM Waagbø R Espe M. Functional amino acids in fish nutrition, health and welfare. Front Biosci. (2015) 8:143–69. doi: 10.2741/757 26709652

[88] Zhang J Liu XS Hu CY Chen XJ Sun X Xu NJ. Physiological and transcriptome analysis of exogenous l-arginine in the alleviation of high-temperature stress in Gracilariopsis lemaneiformis. Front Mar Sci. (2021) 8:784586. doi: 10.3389/fmars.2021.784586

[89] Hoseini SM Khan MA Yousefi M Costas B. Roles of arginine in fish nutrition and health: insights for future researches. Rev Aquacult. (2020) 12:2091–108. doi: 10.1111/raq.12424

[90] Wu GY Baze FW Burghardt RC Johnson GA Kim SW Knabe DA. Proline and hydroxyproline metabolism: implications for animal and human nutrition. Amino Acids. (2011) 40:1053–63. doi: 10.1007/s00726-010-0715-z PMC3773366 20697752

[91] Zhang Z Sun Y Zeng Y Cui N Li B Zhang W. Elucidating the hepatoprotective mechanisms of cholic acid against CCl4-Induced acute liver injury: A transcriptomic and metabolomic study. J Ethnopharmacol. (2024) 328:118052. doi: 10.1016/j.jep.2024.118052 38518967

[92] De Santi C Nally FK Afzal R Duffy CP Fitzsimons S Annett SL. Enhancing arginase 2 expression using target site blockers as a strategy to modulate macrophage phenotype. Mol Ther Nucleic Acids. (2022) 29:643–55. doi: 10.1016/j.omtn.2022.08.004 PMC9424864 36090747

[93] Dowling JK Afzal R Gearing LJ. Mitochondrial arginase-2 is essential for IL-10 metabolic reprogramming of inflammatory macrophages. Nat Commun. (2021) 12:1460. doi: 10.1038/s41467-021-21617-2 33674584 PMC7936006

