# Supplementary material for: Comprehensive metabolomics and transcriptomics analyses investigating the regulatory effects of different sources of dietary astaxanthin on the antioxidant and immune functions of commercial-sized rainbow trout
Source: Front Immunol. 2024 Sep 26;15:1408168. doi: 10.3389/fimmu.2024.1408168 (PMC11464810; doi:10.3389/fimmu.2024.1408168)
Supplement: Supplementary file 1 [file Table_1.docx]

**Supplementary table 1**

Elution gradient of mobile phase

| **Times（min）** | **Mobile phase A (%)** | **Mobile phase B (%)** |
| --- | --- | --- |
| 0.01 | 95 | 5 |
| 2.00 | 95 | 5 |
| 4.00 | 70 | 30 |
| 8.00 | 50 | 50 |
| 10.00 | 20 | 80 |
| 14.00 | 0 | 100 |
| 15.00 | 0 | 100 |
| 15.10 | 95 | 5 |
| 16.00 | 95 | 5 |

**Supplementary table 2**

Parameters of ion scanning mass spectrometry

| **Parameters** | **Positive ion** | **Negative ion** |
| --- | --- | --- |
| Nebulizer Gas (GS1, PSI) | 55 | 55 |
| Auxiliary Gas (GS2, PSI) | 55 | 55 |
| Curtain Gas (CUR, PSI） | 35 | 35 |
| Ion Source Temperature (℃) | 550 | 550 |
| Ion Spray Voltage (V) | 5500 | -4500 |
| Delustering Potential (DP, V) | 80 | -80 |
| Mass Scan Range (TOF MS scan) | 100-1000 | 100-1000 |
| Collision Energy (TOF MS scan, eV) | 10 | -10 |
| Mass Scan Range (Product Ion scan) | 40-1000 | 40-1000 |
| Collision Energy (Product Ion scan, eV） | 35 | -35 |
| Interface Heater Temperature (℃) | 550 | 550 |

**Supplementary table 3**

Specific primers used for qRT-PCR

| **Primers** | **Forward primer sequence (5' to 3')** | **Reverse primer sequence (5' to 3')** | **NCBI accession number or reference** |
| --- | --- | --- | --- |
| *NLRC3* | GCACACTCTGGACCTACGA | GCTCCATCCATTCCCAGAGAG | XM_036946482.1 |
| *A2M* | GTCAAGCTTCTCTCCGGCTT | CCTTCTGCTTGAGCCCATCT | XM_036961906.1 |
| *ITGB1* | GGCAGAGGACTATCCCATCG | AGGAGCCGAAACCGATCTTG | NM_001034987.1 |
| *CXCR2* | GGACATGTAAAGCCAGCTCATGG | AGGGTCAGGGAGAAGAGGAGGTC | Xu et al. (2014) |
| *β-actin* | GCTTCTCAGTCTCATTGG | ACCGTTCCAGTTGTGTATA | Long et al. (2015) |

**Supplementary table 4**

Differential metabolites in the serum of *O. mykiss* between the NA and HPA groups

| Differential metabolites | VIP ^a^ | *P* value ^b^ | FC ^c^ | Levels ^d^ |
| --- | --- | --- | --- | --- |
| Monoethylhexyl phthalic acid | 4.69 | 4.69×10^-2^ | 0.86 | Down |
| PE(20:3(8Z,11Z,14Z)/22:6(4Z,7Z,10Z,13Z,16Z,19Z)) | 4.33 | 3.43×10^-2^ | 0.31 | Down |
| Hydantoin-5-propionic acid | 1.03 | 3.23×10^-2^ | 0.85 | Down |
| PC(18:1(11Z)/18:1(11Z)) | 47.11 | 4.13×10^-8^ | 12.37 | Up |
| PI(16:2(9Z,12Z)/22:3(10Z,13Z,16Z)) | 2.49 | 1.58×10^-4^ | 13.71 | Up |
| Astaxanthin | 2.07 | 9.72×10^-3^ | 8.60 | Up |
| gamma-Glutamylleucine | 1.48 | 1.60×10^-2^ | 1.24 | Up |
| Palmitoleic acid | 1.23 | 1.65×10^-4^ | 3.58 | Up |
| Stachyose | 1.20 | 2.94×10^-2^ | 2.23 | Up |
| Linalyl oxide | 1.02 | 8.95×10^-5^ | 2.40 | Up |
| Palmitic acid | 1.00 | 2.64×10^-3^ | 2.56 | Up |

^a^ Variable importance in the projection (VIP) was acquired from the OPLS-DA model with a threshold of 1.0.

^b^ *P* values were calculated from a two-tailed Student’s t-test.

^c^ FC = fold change, mean value of peak area obtained from the HPA group /mean value of peak area obtained from the NA group. If the FC value is > 1, it means that metabolites in the HPA are more than in the NA.

^d^ Up: an increase in the HPA group, down: a decrease in the HPA group.

**Supplementary table 5**

Differential metabolites in the serum of *O. mykiss* between the NA and PRA groups

| Differential metabolites | VIP ^a^ | *P* value ^b^ | FC ^c^ | Levels ^d^ |
| --- | --- | --- | --- | --- |
| PE(20:3(8Z,11Z,14Z)/22:6(4Z,7Z,10Z,13Z,16Z,19Z)) | 3.81 | 1.57×10^-2^ | 0.22 | Down |
| Aniline | 1.48 | 1.94×10^-2^ | 0.27 | Down |
| 3-Methylhistamine | 1.31 | 8.50×10^-4^ | 0.30 | Down |
| Histamine | 1.16 | 1.17×10^-3^ | 0.31 | Down |
| LysoPC(20:0/0:0) | 2.28 | 1.81×10^-2^ | 0.38 | Down |
| (3Z,6Z)-3,6-Nonadienal | 1.24 | 1.20×10^-2^ | 2.11 | Up |
| Dihydrocarvone | 1.08 | 3.32×10^-3^ | 2.28 | Up |
| Proline betaine | 1.26 | 2.80×10^-2^ | 2.45 | Up |
| Dihydro-5-pentyl-2(3H)-furanone | 1.11 | 1.20×10^-3^ | 2.63 | Up |
| Palmitoleic acid | 1.12 | 3.60×10^-5^ | 4.64 | Up |
| 4-Ethylphenol | 6.98 | 5.30×10^-6^ | 10.65 | Up |
| Glycyl-glycine | 1.05 | 2.10×10^-6^ | 14.47 | Up |
| PI(16:2(9Z,12Z)/22:3(10Z,13Z,16Z)) | 2.08 | 3.30×10^-7^ | 15.98 | Up |
| PC(18:1(11Z**)/**18:1(11Z)) | 44.40 | 1.20×10^-5^ | 20.04 | Up |
| L-Histidinol | 9.17 | 5.00×10^-7^ | 20.24 | Up |
| Astaxanthin | 1.60 | 4.30×10^-2^ | 2.71 | Up |

^a^ Variable importance in the projection (VIP) was acquired from the OPLS-DA model with a threshold of 1.0.

^b^ *P* values were calculated from a two-tailed Student’s t-test.

^c^ FC = fold change, mean value of peak area obtained from the PRA group /mean value of peak area obtained from the NA group. If the FC value is > 1, it means that metabolites in the PRA are more than in the NA.

^d^ Up: an increase in the PRA group, down: a decrease in the PRA group.

**Supplementary table 6**

Differential metabolites in the serum of *O. mykiss* between the NA and SA groups

| Differential metabolites | VIP ^a^ | *P* value ^b^ | FC ^c^ | Levels ^d^ |
| --- | --- | --- | --- | --- |
| 5,6-Dihydrouridine | 2.56 | 3.17×10^-2^ | 0.15 | Down |
| Aniline | 2.09 | 2.03×10^-2^ | 0.26 | Down |
| 3-Methylhistamine | 1.73 | 1.55×10^-3^ | 0.30 | Down |
| Histamine | 1.53 | 2.10×10^-3^ | 0.32 | Down |
| LysoPC(16:1(9Z)/0:0) | 2.41 | 2.80×10^-2^ | 0.52 | Down |
| m-Coumaric acid | 2.58 | 4.82×10^-2^ | 1.46 | Up |
| Dihydro-5-pentyl-2(3H)-furanone | 1.21 | 1.72×10^-2^ | 2.26 | Up |
| Linalyl oxide | 1.10 | 2.60×10^-5^ | 2.69 | Up |
| 2-ene-Valproic acid | 1.10 | 3.30×10^-6^ | 2.88 | Up |
| Palmitoleic acid | 1.22 | 3.60×10^-4^ | 3.69 | Up |
| 4-Ethylphenol | 9.15 | 3.40×10^-8^ | 10.86 | Up |
| PC(18:1(11Z)/18:1(11Z)) | 51.38 | 1.00×10^-4^ | 14.83 | Up |
| Glycyl-glycine | 1.43 | 3.70×10^-11^ | 15.41 | Up |
| PI(16:2(9Z,12Z)/22:3(10Z,13Z,16Z)) | 2.78 | 9.40×10^-6^ | 16.49 | Up |
| Cis-zeatin | 1.36 | 1.10×10^-9^ | 18.90 | Up |
| L-Histidinol | 11.74 | 4.30×10^-10^ | 19.86 | Up |
| 2-Oxoarginine | 1.04 | 3.50×10^-10^ | 33.58 | Up |
| Astaxanthin | 1.42 | 3.58×10^-2^ | 2.49 | Up |

^a^ Variable importance in the projection (VIP) was acquired from the OPLS-DA model with a threshold of 1.0.

^b^ *P* values were calculated from a two-tailed Student’s t-test.

^c^ FC = fold change, mean value of peak area obtained from the SA group /mean value of peak area obtained from the NA group. If the FC value is > 1, it means that metabolites in the SA are more than in the NA.

^d^ Up: an increase in the SA group, down: a decrease in the SA group.

**Supplementary table 7**

Differential metabolites in the serum of *O. mykiss* between the HPA and PRA groups

| Differential metabolites | VIP ^a^ | *P* value ^b^ | FC ^c^ | Levels ^d^ |
| --- | --- | --- | --- | --- |
| Phenol glucuronide | 1.17 | 1.66×10^-2^ | 0.28 | Down |
| Stachyose | 1.54 | 4.13×10^-3^ | 0.29 | Down |
| Astaxanthin | 1.76 | 3.70×10^-2^ | 0.32 | Down |
| Maltotriose | 1.57 | 2.62×10^-2^ | 0.38 | Down |
| Arachidic acid | 1.39 | 2.27×10^-2^ | 0.49 | Down |
| Sphingosine | 1.87 | 4.41×10^-2^ | 0.62 | Down |
| 4-phenylboronic acid-O-sulphate | 1.38 | 4.66×10^-2^ | 0.64 | Down |
| LysoPC(18:3(6Z,9Z,12Z)) | 2.28 | 3.43×10^-2^ | 0.67 | Down |
| Fexofenadine | 1.61 | 5.00×10^-3^ | 0.68 | Down |
| LysoPC(22:5(7Z,10Z,13Z,16Z,19Z)) | 5.79 | 2.65×10^-2^ | 0.72 | Down |
| Diisobutyl phthalate | 1.59 | 3.83×10^-2^ | 1.16 | Up |
| 2-Phenylacetamide | 1.05 | 2.34×10^-2^ | 1.18 | Up |
| Hydantoin-5-propionic acid | 1.35 | 2.18×10^-2^ | 1.20 | Up |
| Dihydro-5-pentyl-2(3H)-furanone | 1.06 | 1.25×10^-2^ | 1.41 | Up |
| Dihydrocarvone | 1.13 | 1.39×10^-2^ | 1.45 | Up |
| PC(18:1(11Z)/18:1(11Z)) | 41.31 | 1.12×10^-2^ | 1.62 | Up |
| 4-Ethylphenol | 8.05 | 1.68×10^-3^ | 2.93 | Up |
| L-Histidinol | 11.55 | 1.80×10^-4^ | 3.91 | Up |
| 2-Oxoarginine | 1.03 | 5.10×10^-5^ | 5.17 | Up |
| Cis-zeatin | 1.43 | 1.20×10^-5^ | 6.15 | Up |
| Glycyl-glycine | 1.53 | 4.00×10^-6^ | 8.26 | Up |

^a^ Variable importance in the projection (VIP) was acquired from the OPLS-DA model with a threshold of 1.0.

^b^ *P* values were calculated from a two-tailed Student’s t-test.

^c^ FC = fold change, mean value of peak area obtained from the PRA group /mean value of peak area obtained from the HPA group. If the FC value is > 1, it means that metabolites in the PRA are more than in the HPA.

^d^ Up: an increase in the PRA group, down: a decrease in the PRA group.

**Supplementary table 8**

Differential metabolites in the serum of *O. mykiss* between the HPA and SA groups

| Differential metabolites | VIP ^a^ | *P* value ^b^ | FC ^c^ | Levels ^d^ |
| --- | --- | --- | --- | --- |
| Arachidic acid | 1.99 | 8.22×10^-3^ | 0.40 | Down |
| Behenic acid | 1.62 | 9.30×10^-4^ | 0.41 | Down |
| Estrone glucuronide | 2.41 | 6.35×10^-3^ | 0.45 | Down |
| Stachyose | 1.54 | 4.69×10^-2^ | 0.49 | Down |
| 3-Dehydroquinate | 1.29 | 6.09×10^-3^ | 0.52 | Down |
| Palmitic acid | 1.39 | 1.22×10^-2^ | 0.57 | Down |
| LysoPC(16:1(9Z)/0:0) | 3.68 | 3.45×10^-2^ | 0.63 | Down |
| N-Carboxyethyl-g-aminobutyric acid | 1.87 | 3.00×10^-3^ | 0.69 | Down |
| 2-Phenylacetamide | 1.77 | 3.71×10^-3^ | 1.17 | Up |
| Hydantoin-5-propionic acid | 2.17 | 7.73×10^-3^ | 1.19 | Up |
| 4-Ethylphenol | 14.04 | 4.00×10^-4^ | 2.99 | Up |
| L-Histidinol | 18.43 | 4.20×10^-5^ | 3.83 | Up |
| 2-Oxoarginine | 1.70 | 3.80×10^-6^ | 5.52 | Up |
| Cis-zeatin | 2.29 | 2.60×10^-8^ | 6.87 | Up |
| L-Glutamine | 1.05 | 2.30×10^-8^ | 7.56 | Up |
| Acetylisoniazid | 1.20 | 5.40×10^-7^ | 7.87 | Up |
| Glycyl-glycine | 2.42 | 2.20×10^-10^ | 8.80 | Up |
| L-2,4-diaminobutyric acid | 1.55 | 1.30×10^-9^ | 270.84 | Up |

^a^ Variable importance in the projection (VIP) was acquired from the OPLS-DA model with a threshold of 1.0.

^b^ *P* values were calculated from a two-tailed Student’s t-test.

^c^ FC = fold change, mean value of peak area obtained from the SA group /mean value of peak area obtained from the HPA group. If the FC value is > 1, it means that metabolites in the SA are more than in the HPA.

^d^ Up: an increase in the SA group, down: a decrease in the SA group.
